# Removal of Selenium Oxyanions from Aqueous Solutions by Ion Exchange: Equilibrium, Kinetics, and Mechanistic Modeling

**DOI:** 10.1021/acsestwater.5c01136

**Published:** 2025-12-23

**Authors:** Z. Zeng, Z. Shen, J. D. Einkauf, A. P. Ladshaw, R. Custelcean, C. Tsouris, S. Yiacoumi

**Affiliations:** † School of Civil and Environmental Engineering, 1372 Georgia Institute of Technology, Atlanta, Georgia 30332, United States; ‡ 6146 Oak Ridge National Laboratory, Oak Ridge, Tennessee 37831, United States

**Keywords:** selenium oxyanions, selenate, selenite, ion exchange, ion-exchange modeling, IRA-900

## Abstract

Selenium (Se) is an essential micronutrient but toxic at high concentrations, posing challenges for water treatment. This study investigated the removal of selenate (SeO_4_^2–^) and selenite (SeO_3_^2–^) using the strong-base anion-exchange resin IRA-900, particularly in the presence of competing sulfate (SO_4_^2–^). The performance of the commercially available resin IRA-900 was systematically investigated. The batch equilibrium behavior was studied in both single- and binary-component systems, and the kinetic behavior was investigated in single-component systems. Results confirmed a selectivity order of SeO_4_^2–^ > SO_4_^2–^ > SeO_3_^2–^, indicating preferential SeO_4_^2–^ removal over competing SO_4_^2–^ but lower affinity for SeO_3_^2–^. The maximum total exchange capacity was determined to be 2.04 mequiv/g. Furthermore, SeO_3_^2–^ uptake was found to be pH-dependent, whereas SeO_4_^2–^ uptake remained stable across a broad pH range. From a modeling perspective, the Law of Mass Action model effectively described equilibrium data, and a transport–reaction modeling framework captured removal kinetics of oxyanions including film and intraparticle diffusion. Finally, X-ray photoelectron spectroscopy confirmed ion exchange between chloride and Se oxyanions as the primary removal mechanism. These findings provide fundamental insights into the removal of Se oxyanions from aqueous solutions by ion exchange.

## Graphical abstract

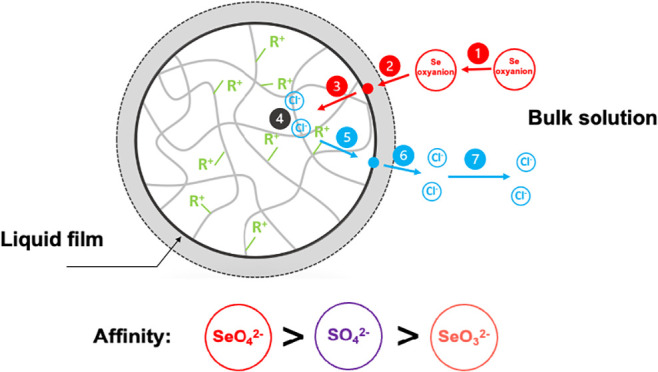

## 1 Introduction

Selenium (Se) is an essential element for most living organisms at trace levels but becomes toxic at higher concentrations. This dual nature of Se, being both essential and toxic within a narrow concentration range (40 to 400 μg/day for humans), has generated considerable scientific and technological interest to monitor and control its environmental concentrations.^1,2^ Se occurs naturally in the earth’s crust (30th most abundant element), primarily in carbonate and phosphate rocks, volcanic and sedimentary soils, and coal, and is redistributed to atmospheric, aquatic, and terrestrial environments through natural and anthropogenic processes.^2,3^ Human activity is a major factor in the mobilization of Se through mining, fossil fuel combustion, oil refining, and agricultural activities.^4^ Advances in water quality and pollution monitoring in recent years have shown that Se is a contaminant of potential environmental concern, prompting the United States Environmental Protection Agency (EPA) to establish stringent regulatory discharge limits of 50 μg/L (ppb) for drinking water and 5 μg/L (ppb) for freshwater systems.^5–8^ While Se can occur in a wide range of chemical species with different oxidation states, in industrial (e.g., fossil fuel power plants) aqueous solutions, it is mostly present as selenite [SeO_3_^2–^, Se (IV)] and selenate [SeO_4_^2–^, Se (VI)] oxyanions,^4,9^ with sulfate (SO_4_^2–^) being one of the most common and abundant competing ions for these species.^2,5,10,11^

Various technologies have been developed for Se removal from aqueous solutions. Physical treatment is primarily based on membrane filtration, e.g., nanofiltration (NF) or reverse osmosis (RO).^12,13^ While these methods can effectively remove Se concentration to below 5 μg/L, they involve high capital, operational, and maintenance costs and typically require pretreatment to remove suspended solids and minimize scaling and fouling.

Sorption, including ion exchange, is one of the approaches developed for removing Se from water, and numerous materials have been reported to be effective for this purpose.^10,14–24^ Table 1 summarizes the results of diverse materials including commercial ion-exchange resins or sorbents, natural iron oxides, and synthetic composites, detailing critical performance characteristics for each material, including its Se uptake capacity alongside its primary advantages and limitations.

**Table 1:** Performance Summary of Selected Sorbents for Se Removal from Water^a^

origin	material	target ions [capacity (mg Se/g)]	advantages	limitations	references
Commercial ion-exchange resins/sorbents	AmberLite IRA-900	SeO_4_^2–^ (*q*_*e*_ = 38.7)	High SeO_4_^2–^ capacity	SeO_4_^2–^ competing with SO_4_^2–^	Tan et al.^10^
			Fast kinetics (∼7 h)	No SeO_3_^2–^ data	
			Efficient regeneration		
			Wide operational pH range for SeO_4_^2–^		
			Beads ensure uniform packing		
			Cost effective		
	AmberLite IRA-400	SeO_3_^2–^ (*q*_*m*_ = 18.5)	Fast uptake (<1 h)	Low capacity	El-Tantawy et al.^14^
			Wide operational pH range	No SeO_4_^2–^ data	
			Efficient regeneration		
	AmberLite IRA-67	SeO_4_^2–^ (*q*_*e*_ = 0.19)	N/A	Extremely low capacity	Erosa et al.^15^
		SeO_3_^2–^ (*q*_*e*_ = 0.1)		Highly pH-dependent	
	FerrIX A33E	SeO_4_^2–^ (N/A)	High removal efficiency	Narrow pH range (4.5–8.5)	Staicu et al.^16^
		SeO_3_^2–^ (N/A)	Removes other toxic metals	Strong SO_4_^2–^ competition	
	Eporasu K-6	SeO_4_^2–^ (*q*_*m*_ = 134)	Efficient regeneration	pH-dependent performance	Nishimura et al.^17^
		SeO_3_^2–^ (N/A)	Higher affinity for SeO_4_^2–^		
	Smopex-103	SeO_4_^2–^ (*q*_*e*_ = 67.1)	High capacities	Poor fixed-bed compatibility	Namara et al.^18^
		SeO_3_^2–^ (*q*_*e*_ = 55.3)	Ultrafast uptake (<1 min)		
Natural	Goethite	SeO_4_^2–^ (*q*_*m*_ = 0.17)	Extremely low-cost	Low capacity	Rovira et al.^19^
		SeO_3_^2–^ (*q*_*m*_ = 0.52)		Slow kinetics (30–50 h)	
	Hematite	SeO_4_^2–^ (*q*_*m*_ = 0.24)		pH-sensitive	
		SeO_3_^2–^ (*q*_*m*_ = 0.39)		Poor fixed-bed compatibility	Martínez et al.^20^
	Magnetite	SeO_4_^2–^ (*q*_*m*_ = 0.25)			
		SeO_3_^2–^ (*q*_*m*_ = 0.22)			
Synthetic	MgAl(MoS_4_)_2_–LDH	SeO_4_^2–^ (*q*_*e*_ = 85)	High capacities	Mo leaching risk	Ma et al.^21^
		SeO_3_^2–^ (*q*_*e*_ = 294)	Extremely fast kinetics	Performance depends on heavy metals	
	FeMgAl(MoS_4_)_2_–LDH	SeO_4_^2–^ (*q*_*m*_ = 92)	High capacities	Mo leaching risk	Liao et al.^22^
		SeO_3_^2–^ (*q*_*m*_ = 301)	Built-in catalyst	Complex synthesis	
				Slower kinetics (600 min)	
	MgAl-MoO_2_S_2_-LDH	SeO_4_^2–^ (*q*_*e*_ = 131)	High capacities	Complex synthesis and the use of formamide	Alam et al.^23^
		SeO_3_^2–^ (*q*_*e*_ = 223)	Maintains efficiency in presence of competing anions	Mo leaching risk	
	Carbon-supported magnetic nanoparticle adsorbent (C-MNA)	SeO_3_^2–^ (*q*_*e*_ = 47.8)	High SeO_3_^2–^ uptake capacity	No SeO_4_^2–^ data	Evans et al.^24^
			Rapid uptake (<30 min)	Narrow pH range	

a A comparison of reported sorption capacities, advantages, and limitations for materials targeting SeO_4_^2–^ and SeO_3_^2–^. Capacities are reported as theoretical maximum (*q*_*m*_) or experimental equilibrium (*q*_*e*_) values in elemental Se per gram of sorbent (i.e., mg Se/g) unless otherwise specified.

Commercially available ion-exchange resins and sorbents exhibit considerable diversity in their performance in Se removal. For instance, advanced fibrous sorbents such as Smopex-103 display high capacities for both SeO_4_^2–^ and SeO_3_^2–^ and demonstrate fast uptake kinetics, reaching equilibrium in under a minute. Their fibrous structure results, however, in poor fixed-bed compatibility, posing a significant challenge for continuous-flow applications.^18^ Other specialized resins show similar trade-offs. Eporasu K-6 offers a remarkably high capacity for SeO_4_^2–^ and efficient regeneration, but its highly pH-dependent performance creates operational uncertainty.^17^ The iron-based composite FerrIX A33E provides high removal efficiency but is severely limited by a narrow pH range and strong competition from SO_4_^2–^.^16^ Meanwhile, other commercial resins are constrained by insufficient capacity; AmberLite IRA-400 has fast uptake but a low capacity and no performance data for SeO_4_^2–^, and AmberLite IRA-67 is largely impractical due to its extremely low capacity and high pH sensitivity.^14,15^

Among natural sorbents, iron oxide minerals are often investigated due to their low cost and abundance. Their application is, however, severely hindered by a combination of low sorption capacities, slow kinetics that require days to reach equilibrium, and a powder morphology unsuitable for packed-bed columns.^19,20^ In addition, synthetic sorbents report notable laboratory performance, which is frequently offset by significant drawbacks. The magnetic nanocomposite C-MNA exhibits high SeO_3_^2–^ capacities of 47.8 mg/g, but its fine particle size limits applicability in packed-bed columns, and it has not demonstrated effectiveness for SeO_4_^2–^ removal. Some dual-mode materials that integrate ion-exchange and redox functions have also been developed. One example is molybdenum-sulfide–intercalated magnesium–aluminum layered double hydroxide [MgAl­(MoS_4_)_2_-LDH], which demonstrated a high capacity for SeO_3_^2–^ (294 mg Se/g via redox-driven chemisorption process) and SeO_4_^2–^ (85 mg Se/g via ion exchange) within 10 min, and shows enhanced removal in the presence of heavy metals such as Hg^2+^ and Cu^2+^, a behavior not observed in conventional ion-exchange systems. Its application is, however, limited by the potential for molybdenum (Mo) leaching and the strong dependence on heavy metals.^21,22^ Building on the same concept, FeMgAl­(MoS_4_)_2_-LDH incorporates iron as an internal catalyst, increasing the SeO_3_^2–^ and SeO_4_^2–^ capacity to 301 mg Se/g and 92 mg Se/g, respectively. Despite its high performance, its practical application is still limited by complex synthesis, reduced effectiveness under alkaline conditions, and the potential for Mo leaching. A more recent molybdenum-oxysulfide–functionalized MgAl-LDH (MgAl-MoO_2_S_2_-LDH) also achieves high capacities for SeO_3_^2–^ and SeO_4_^2–^ (223 mg Se/g and 131 mg Se/g, respectively) and maintains performance in the presence of competing ions such as SO_4_^2–^, HCO_3_^–^, and Cl^–^, although its synthesis remains complex and in requires formamide.^23^ Simultaneously, methods relying solely on chemical reduction, using reducing agents such as zerovalent iron or electrochemical processes have been examined, but these approaches are hindered by slow reaction kinetics at low Se concentrations, resulting in long residence times and high cost of electrode materials.^25,26^

Among these materials and methods, AmberLite IRA-900, a strong-base anion-exchange resin, stands out due to its well-balanced combination of critical features. It exhibits a high experimentally verified uptake capacity for SeO_4_^2–^ (38.7 mg Se/g), which is the prevalent Se oxyanion in industrial aqueous systems.^10^ In addition, IRA-900 demonstrates moderate uptake kinetics (7 h) as well as a broad effective pH range (from strongly acidic to strongly alkaline, pH 0–13). Furthermore, its efficient regeneration under mildly acidic conditions further enhances its operational viability.^10^ In a continuous flow operation, the spherical bead form of IRA-900 also contributes to uniform and predictable hydraulic behavior, reducing pressure drop issues and facilitating effective operation in fixed-bed column processes. The resin’s performance for SeO_3_^2–^ removal and a direct experimental comparison of its relative affinities for SeO_4_^2–^, SeO_3_^2–^, and SO_4_^2–^ remain, however, to be determined.^10^

Another chemical treatment method, precipitation (coprecipitation), was sometimes employed as a pretreatment step to remove both Se and key competing ions before a final ion-exchange polishing stage. Barium chloride (BaCl_2_) is frequently applied because barium ions react with SO_4_^2–^ to form insoluble barium sulfate (BaSO_4_), and with SeO_4_^2–^ to precipitate sparingly soluble barium selenate (BaSeO_4_).^16,17^ As a result, BaCl_2_ pretreatment prior to using FerrIX A33E increased total Se removal from industrial effluents from below 3 to 80%.^16^ Similarly, once most of SO_4_^2–^ has been removed as BaSO_4_, Eporasu K-6 reduced Se (VI) from 780 ppm to below 0.1 ppm.^17^ Single-step precipitation has also been explored. Guanidinium-based ligands were shown to remove SeO_4_^2–^ and SO_4_^2–^ through the formation of highly insoluble SO_4_^2–^/SeO_4_^2–^ crystalline solids, achieving near quantitative (>99%) SeO_4_^2–^ removal even at subppb concentrations.^11^ Their applications, however, require strict control to eliminate biotoxicity concerns, and more broadly, most coprecipitation methods offer limited selectivity between target and competing ions.

In previous studies, the Langmuir and Freundlich models were applied to characterize sorption equilibrium in aqueous solutions. Although commonly employed in adsorption processes, these models are fundamentally empirical when applied to ion-exchange processes.^10,18,27–29^ The absence of comprehensive physicochemical foundations underlying these models can result in reduced accuracy under certain conditions. Kinetic models, such as the pseudo-first order and pseudo-second order kinetic models, are generally used to analyze dynamic experimental data.^10,29–32^ None of these models, however, considers mass transfer mechanisms, such as film diffusion or intraparticle diffusion, which can significantly affect ion-exchange kinetics. In addition, empirical models neglect the true mechanistic nature of transport processes, especially under conditions where basic model assumptions no longer hold.^33,34^ Such limitations highlight the need for using more comprehensive models or combining empirical with mechanistic models to ensure both deeper understanding of the ion-exchange process and accurate description.

This study focuses on mechanistic understanding and model development for Se oxyanions removal in batch ion-exchange systems using a commercially available resin, rather than on developing and modifying materials for enhancing Se capacity. AmberLite IRA-900 is selected as the benchmark strong-base anion-exchange resin due to its balanced properties and its well-documented performance. Prior work, particularly the study by Tan et al., provided valuable insights into its performance for SeO_4_^2–^ removal in the presence of SO_4_^2–^, yet key knowledge gaps remain.^10^ The resin’s performance for SeO_3_^2–^ has not been systematically studied, its selectivity among SeO_4_^2–^, SeO_3_^2–^, and SO_4_^2–^ has not been experimentally quantified, and the influence of solution pH has not been fully examined. Previous studies also relied primarily on empirical models. The present study addresses these gaps by providing a more comprehensive, mechanistically grounded investigation of SeO_4_^2–^ and SeO_3_^2–^ removal and their competition with SO_4_^2–^ by IRA-900, including equilibrium behavior and selectivity in single- and multicomponent systems, uptake kinetics, and pH effects. SO_4_^2–^ was selected as the primary competitor as it dominates in SO_4_^2–^-rich industrial (i.e., fossil fuel power plants) aqueous systems, where S:Se ratios can exceed 1000:1.^11^ Temperature effects were not examined in this study because IRA-900 performance varies minimally between 20 and 30 °C,^10^ thus all experiments were conducted at room temperature (20 °C). To provide deeper mechanistic interpretation, this works also develops and applies Law of Mass Action (LMA) and mass-transfer-based models. Finally, X-ray Photoelectron Spectroscopy (XPS) was employed to provide direct evidence of the ion-exchange mechanism at an elemental level. Together, these components build upon prior studies to deliver a more comprehensive framework for understanding and quantitatively describing the performance of IRA-900, supporting the design and optimization of industrial-scale anion-exchange systems.

## 2 Materials and Methods

### 2.1 Materials

AmberLite IRA-900 [chloride (Cl^–^) form] is a strong-base, microporous anion-exchange resin composed of a styrene-divinylbenzene matrix functionalized with trimethylammonium groups. The resin, obtained from Sigma-Aldrich as tan spherical beads with particle size of 640–800 μm, was used as received without additional pretreatment. Its water content is 60% according to the manufacturer.^35^ Analytical-grade chemicals were also purchased from Sigma-Aldrich. SeO_3_^2–^ and SeO_4_^2–^ were supplied as sodium selenite (Na_2_SeO_3_) or sodium selenate (Na_2_SeO_4_), respectively, and SO_4_^2–^ was added as potassium sulfate (K_2_SO_4_). Single- and multicomponent solutions were prepared in Milli-Q water (18 MΩ·cm). Solution pH was adjusted using hydrochloric acid (HCl, 1 N) and sodium hydroxide (NaOH, prepared as a 1 M solution in Milli-Q water).

### 2.2 Batch Experiments

Single-component (SeO_4_^2–^, SeO_3_^2–^, or SO_4_^2–^) and multicomponent (SeO_4_^2–^/SO_4_^2–^ and SeO_3_^2–^/SO_4_^2–^) batch equilibrium experiments were performed. All solutions were prepared in Milli-Q water without further pH adjustment, yielding near-neutral conditions (pH = 7 ± 0.5) at equilibrium. For single-component equilibrium experiments, 10 mM SeO_4_^2–^, 10 mM SO_4_^2–^, and 5 mM SeO_3_^2–^ solutions were prepared in 100 mL solution volume using 125 mL Erlenmeyer flasks. Resin dosages were selected based on preliminary results to ensure adequate coverage of both low- and high-capacity regions of the isotherms and reliable estimation of equilibrium parameters across a wide range of conditions: 6, 8, 10, 15, 20, 30, and 50 g/L for SeO_4_^2–^; 2.5, 5, 8, 11, 15, 20, 35, and 50 g/L for SO_4_^2–^; and 2, 3, 5, 7.5, 12.5, 17.5, 25, and 75 g/L for SeO_3_^2–^. Given that SO_4_^2–^ typically dominates in industrial aqueous solutions where sulfur (S) to Se molar ratios exceed 1000:1,^11^ binary equilibrium experiments were designed to reflect realistic environmental conditions. Solutions contained a fixed SO_4_^2–^ concentration of 2.5 mM, while SeO_4_^2–^ or SeO_3_^2–^ was varied at 2.5, 0.25, 0.025, and 0.0025 mM (Se:S molar ratios of 1, 10, 100 and 1000). These tests used 20 g/L IRA-900 in 50 mL solutions, a dosage selected based on preliminary screening, ensuring sufficient removal without excessive resin use. To examine pH effects, equilibrium experiments were conducted at initial pH values of 2, 4, 6, 8, and 10 using a constant resin dosage of 20 g/L in 100 mL solutions with 1.5 mM Se. The pH was adjusted using 0.1 or 1 M NaOH and HCl solutions, and the final pH was recorded after 24 h of contact. All batch equilibrium experiments were performed in a shaker (Cole-Parmer WB-200) at 20 °C for 24 h and 160 rpm to provide adequate mixing while avoiding bead damage.

For kinetic experiments, SeO_4_^2–^, SeO_3_^2–^, and SO_4_^2–^ uptake was monitored over time for initial concentrations of 1, 5, and 10 mM and 20 g/L resin dosage in 100 mL solutions. Flasks were maintained at 160 rpm and 20 °C, and 0.2 mL samples were periodically collected from the supernatant over 3 h to track Se uptake.

### 2.3 Analytical Methods

Liquid aliquots were filtered through 0.02-μm syringe filters to remove suspended solids and diluted with 2% HNO_3_ before analysis by inductively coupled plasma mass spectrometry (ICP-MS, Thermo Scientific iCAP TQ with an Elemental Scientific prepFAST M5 sampling and autodilution system) to measure Se and S concentrations. The morphology of IRA-900 was examined using scanning electron microscopy (SEM, Hitachi SU8010) under secondary electron imaging (SEI) mode at 6 kV to observe surface features at various magnifications. X-ray photoelectron spectroscopy (XPS, Thermo Scientific K-Alpha) was used to compare pristine IRA-900 and resins loaded with SeO_4_^2–^, SeO_3_^2–^, or an equimolar SeO_4_^2–^/SeO_3_^2–^ mixture. Resins for XPS characterization were prepared from batch experiments, performed in the shaker at 20 °C for 24 h and 160 rpm, using 20 g/L resin dosage in 100 mL solutions of 20 mM total Se, and then dried in a vacuum oven at 60 °C overnight. Survey scans and core-level spectra for Se 3d, Cl 2p, N 1s, and O 1s were collected.

### 2.4 Data Analysis Methods

#### 2.4.1 Determination of Sorption Capacity and Removal Efficiency

The sorption capacity of oxyanions onto IRA-900 resin in batch experiments was calculated by eqs 1 and 2, respectively, and the removal efficiency (*RE*) was calculated by eq 3

1 $$q_{e}=\frac{\left(C_{0}-C_{e}\right)V}{W}$$

2 $$q=\frac{\left(C_{0}-C_{b}\right)V}{W}$$

3 $$\mathrm{RE}=\frac{C_{0}-C_{e}}{C_{0}}$$

where *q*_*e*_ and *q* are the sorption capacity at equilibrium and at any given time (*t*, min), respectively, mmol/g or meq/g (milliequivalent/g); *C*_*e*_ and *C*_*b*_ are the oxyanion concentrations in the bulk solution at equilibrium and at any given time (*t*), respectively, mM (mmol/L) or meq/L (milliequivalent/L); *C*_0_ is the initial oxyanion concentration of the bulk solution, mM (mmol/L) or meq/L; *W* is the resin dosage, g; *V* is the volume of the solution, L.

#### 2.4.2 Numerical Models

##### 2.4.2.1 Equilibrium Models

Ion-exchange equilibria can be described with heterogeneous sorption models or models based on LMA, which account not only for the stoichiometry of ion-exchange reactions, but also for the nonideality in the solution/resin phases caused by the ion–solvent and ion–ion interactions. Compared to the Langmuir and Freundlich models, LMA models can better represent the physicochemical phenomena of the process, and thus more accurately describe ion-exchange processes. In this study, IRA-900 resin comes in the form of Cl^–^. As such, the ion-exchange reaction can be exemplified by the exchange between SeO_4_^2–^ and Cl^–^, expressed as follows

4 $$2\overset{®}{R^{+}\;{\mathrm{Cl}}^{-}\;}+{\mathrm{SeO}}_{4}^{2-}\left(\mathrm{aq}\right)↔\overset{®}{{{(R}^{+}\;)}_{2}\;{\mathrm{SeO}}_{4}^{2-}\;}+2{\mathrm{Cl}}^{-}\left(\mathrm{aq}\right)$$

where $\overset{®}{R^{+}\;}$ denotes the species in the resin phase. The LMA equations used in this study are summarized in Table 2.^36–38^

**Table 2:** Equations and Parameter Descriptions for LMA Models That were Implemented in Simulations^a^

models	equations	notes
LMA (nonideal)	5 $K_{A}^{B}\left(T\right)={\left(\frac{\overset{®}{\gamma_{B}\;}q_{B}}{\gamma_{B}C_{B}}\right)}^{z_{A}}{\left(\frac{\gamma_{A}C_{A}}{\overset{®}{\gamma_{A}\;}q_{A}}\right)}^{z_{B}}$	*K*_*A*_^*B*^: Thermodynamic equilibrium constant for *A* exchanged by *B* (L/g for *Z*_*A*_ = 1 and *Z*_*B*_ = 2)
		*k*_*A*_^*B*^: Selectivity coefficient for A exchanged by *B* (L/g for *Z*_*A*_ = 1 and *Z*_*B*_ = 2)
LMA (ideal)	6 $k_{A}^{B}\left(T\right)={\left(\frac{q_{B}}{C_{B}}\right)}^{z_{A}}{\left(\frac{C_{A}}{q_{A}}\right)}^{z_{B}}$	γ̅_*i*_,γ_*i*_: Activity coefficient in resin/solution
		*q*_*i*_: molality in the resin phase (eq/g)
		*C*_*i*_: molarity in the solution phase (eq/L)
Wilson	7 $\ln\overset{®}{\gamma_{A}\;}=-\ln\left(y_{A}+y_{B}\Lambda_{\mathrm{AB}}\right)+y_{B}\left(\frac{\Lambda_{\mathrm{AB}}}{y_{A}+y_{B}\Lambda_{\mathrm{AB}}}-\frac{\Lambda_{\mathrm{BA}}}{y_{A}\Lambda_{\mathrm{BA}}+y_{B}}\right)$	*y*_*A*_,*y*_*B*_: ionic fraction of *A*/*B* in exchanger phase
	8 $\ln\overset{®}{\gamma_{B}\;}=-\ln\left(y_{A}\Lambda_{\mathrm{BA}}+y_{B}\right)-y_{A}\left(\frac{\Lambda_{\mathrm{AB}}}{y_{A}+y_{B}\Lambda_{\mathrm{AB}}}-\frac{\Lambda_{\mathrm{BA}}}{y_{A}\Lambda_{\mathrm{BA}}+y_{B}}\right)$	Λ_*AB*_, Λ_*BA*_: binary interaction parameters
	9 $y_{A}+y_{B}=1$	
Extended Debye–Hückel	10 $I=\frac{1}{2}\sum_{i=1}^{n}z_{i}^{2}m_{i}$	*I*: ionic strength (mol/L)
		*m*_*i*_: concentration of species *i*
	11 $\ln\gamma_{i}=-\frac{A_{\gamma }z_{i}^{2}\sqrt{I}}{1+\beta a\sqrt{I}}$	*A*_γ_ ≈ 0.51 for water@25 °C
		β ≈ 0.33 for water@25 °C
		*a*: ion size parameter (Å)

a Cl^–^/SeO_4_^2–^, Cl^–^/SeO_3_^2–^, and Cl^–^/SO_4_^2–^ chemical systems are studied in this research. In the equations, *A* refers to Cl^–^ and *B* refers to the target ions in single-component systems exchanged with Cl^–^ (i.e., SeO_4_^2–^, SeO_3_^2–^, or SO_4_^2–^ in this study).

The thermodynamic equilibrium constant (*K*_*A*_^*B*^) was used to define the equilibrium relationship of reaction (eq 5). *K*_*A*_^*B*^ is expressed as a function of the activities (*a̅*_*i*_,*a*_*i*_) of the two ions and is dependent only on the reaction temperature. Although the activities cannot be directly measured through experimental methods, they can be calculated using the species concentrations and activity coefficients in resin phase (*q*_*i*_,*γ̅*_*i*_) and bulk solution (*C*_*i*_ *,γ*_*i*_) phase, respectively. A variety of empirical and semiempirical equations can be found in the literature to estimate activity coefficients.^36^ In this study, the Wilson model was adopted for the estimation of γ̅_*i*_ in the resin phase (eqs 7–9),^39^ and the Extended Debye–Hückel model was adopted to calculate γ_*i*_ in the solution phase (eqs 10 and 11).^36^ Note that one way to simplify the model is by assuming ideal solutions in both resin and bulk phases. As shown in eq 6, the selectivity coefficient (*k*_*A*_^*B*^) is used to describe the equilibrium relationship with the assumption that the activity coefficients are equal to 1.

In this work, both ideal and nonideal LMA models were implemented, and the corresponding equilibrium parameters (*K*_*A*_^*B*^,Λ_*AB*_,Λ_*BA*_,*k*_*A*_^*B*^) were optimized by minimizing the objective function (eq 12), where *F*_obj_ denotes the objective function, *y*_*i*_^exp^ is the experimental value, *y*_*i*_^model^ is the model-predicted value, *n* is the total number of data points, and *i* is the index of the data points.^40^ The optimization was achieved using the Python library Pyomo and solved using the Interior Point OPTimizer (IPOPT), a nonlinear programming solver.^40^ The convergence threshold was set as 10^–8^, and parameter ranges were restricted to physically meaningful bounds (*K*_*A*_^*B*^,*k*_*A*_^*B*^ ∈ [0,1200], Λ_*AB*_,Λ_*BA*_ ∈ [0,5]) to avoid nonphysical solutions and overfitting. Across all runs, the solver consistently converged within the prescribed threshold, and multiple initial guesses yielded consistent parameter estimation, indicating the robustness of the optimization. The separation factor, defined by eq 13, is a metric that reflects the selectivity for one species over another. For instance, α_*A*_^*B*^ larger than 1 implies that *B* is more favored than *A*. Specifically, the separation factor for SO_4_^2–^ over SeO_4_^2–^ can be estimated through eq 14 (assuming ideal solutions).

12 $$F_{\mathrm{obj}}=\sum_{i}^{n}{\left(y_{i}^{\exp}-y_{i}^{\mathrm{model}}\right)}^{2}$$

13 $$\alpha_{A}^{B}=\left(\frac{q_{B}}{C_{B}}\right)\left(\frac{C_{A}}{q_{A}}\right)$$

14 $${\alpha_{{\mathrm{SeO}}_{4}^{2-}}^{{\mathrm{SO}}_{4}^{2-}}=k}_{{\mathrm{SeO}}_{4}^{2-}}^{{\mathrm{SO}}_{4}^{2-}}=\left(\frac{q_{{\mathrm{SO}}_{4}^{2-}}}{C_{{\mathrm{SO}}_{4}^{2-}}}\right)\left(\frac{C_{{\mathrm{SeO}}_{4}^{2-}}}{q_{{\mathrm{SeO}}_{4}^{2-}}}\right)=\frac{k_{{\mathrm{Cl}}^{-}}^{{\mathrm{SO}}_{4}^{2-}}}{k_{{\mathrm{Cl}}^{-}}^{{\mathrm{SeO}}_{4}^{2-}}}$$

##### 2.4.2.2 Kinetic Models

The ion exchange uptake mechanisms, as illustrated in Figure 1, can be summarized by seven steps:^41^ (1) diffusion of target ions (SeO_4_^2–^) in the bulk solution surrounding the resin/solution interface, (2) diffusion of SeO_4_^2–^ through the liquid film into the resin matrix, (3) diffusion of SeO_4_^2–^ within the resin matrix, (4) the ion exchange reaction, (5) diffusion of the exchanged ions (Cl^–^) within the resin matrix, (6) diffusion of Cl^–^ into the bulk solution through the liquid film, and (7) diffusion of Cl^–^ in the bulk phase. The rate-limiting steps usually involve transport (step 2: film diffusion and step 3: intraparticle diffusion) and chemical reaction (step 4). In this study, both transport and reaction processes were considered in the modeling.

**Figure 1:**
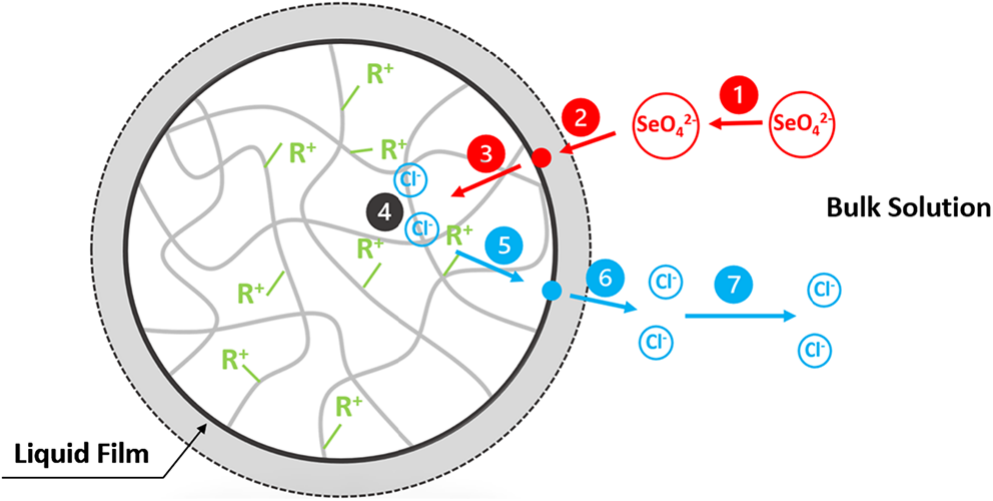
Illustration of the uptake mechanisms for SeO_4_^2–^ into an IRA-900 resin.^41^

Several assumptions were made to build up the kinetic equations. First, the resins are spherical in shape and their volume is constant during the entire process. The experiments were conducted isothermally in a completely mixed system, such that the energy conservation was not considered. Furthermore, the effect of cation permeations (Donnan invasions) is negligible.^37^ The differential equations and initial/boundary conditions used in this study are listed in Table 3.^41,42^

**Table 3:** Macroscale and Microscale Modeling of Mass Transfer Processes, with SeO_4_^2–^ Used as the Representative Species in the Equations^41^

models	equations		notes
macroscale mass transfer	15 $\frac{dC_{b,{\mathrm{SeO}}_{4}^{2-}}}{\partial t}=-\frac{3M}{r_{p}\rho_{p}V}k_{m}\left(C_{b,{\mathrm{SeO}}_{4}^{2-}}-C_{p,{\mathrm{SeO}}_{4}^{2-}|r=r_{p}}\right)$		*M:* Total resin mass (g)
			*V:* Total volume of solution (mL)
			*r*_*p*_ *:* Resin radius (cm)
			*ρ*_*p*_ *:* Resin density (g/cm^3^)
			*k*_*m*_ *:* Film transfer rate (cm/min)
	16 $C_{b,{\mathrm{SeO}}_{4}^{2-}}+C_{b,{\mathrm{Cl}}^{-}}=C_{T}$		*C*_*T*_ *:* Total ionic conc. (eq/mL)
microscale mass transfer	17 $\varepsilon_{p}\frac{\partial C_{p,{\mathrm{SeO}}_{4}^{2-}}}{\partial t}+\rho_{p}\frac{\partial q_{{\mathrm{SeO}}_{4}^{2-}}}{\partial t}=\frac{1}{r^{2}}\frac{\partial }{\partial r}\cdot \left(r^{2}\varepsilon_{p}D_{p}\frac{\partial C_{p,{\mathrm{SeO}}_{4}^{2-}}}{\partial r}\right)$		ε_*p*_ *:* Resin porosity (cm)
			*D*_*p*_ *:* Pore diffusivity (cm^2^/min)
	18 $\frac{dq_{{\mathrm{SeO}}_{4}^{2-}}}{dt}=k_{f}C_{p,{\mathrm{SeO}}_{4}^{2-}}q_{{\mathrm{Cl}}^{-}}^{2}-k_{r}{C_{p,{\mathrm{Cl}}^{-}}^{2}q}_{p,{\mathrm{SeO}}_{4}^{2-}}$		*k*_*f*_: Forward reaction rate constant (eq/g·eq/mL·min)^−1^
			*k*_*r*_: Reverse reaction rate constant (eq/mL)^−2^ (min)^−1^
	19 $q_{{\mathrm{SeO}}_{4}^{2-}}+q_{{\mathrm{Cl}}^{-}}=q_{T}$		*q*_*T*_: Total exchange capacity (eq/g)
	20 $$\overset{®}{q_{{\mathrm{SeO}}_{4}^{2-}}}=\frac{3}{r_{p}^{3}}\int_{0}^{r_{p}}q_{{\mathrm{SeO}}_{4}^{2-}}r^{2}dr$$		$\overset{®}{q_{{\mathrm{SeO}}_{4}^{2-}}}$ : Average exchange capacity within the resin (eq/g)
	$K=\frac{k_{f}}{k_{r}}$		*K*: Reaction equilibrium constant (g/mL)
	21 $\mathrm{initial}\;\mathrm{condition}:C_{p,{\mathrm{SeO}}_{4}^{2-}}=0,@t=0,0\leq r\leq r_{p}$		
	22 $\mathrm{boundary}\;\mathrm{conditions}:\frac{\partial C_{p,{\mathrm{SeO}}_{4}^{2-}}}{\partial r}=0,@r=0$		
	23 $\varepsilon_{p}D_{p}\frac{\partial C_{p,{\mathrm{SeO}}_{4}^{2-}}}{\partial r}=k_{m}\left(C_{b,{\mathrm{SeO}}_{4}^{2-}}-C_{p,{\mathrm{SeO}}_{4}^{2-}|r=r_{p}}\right),@r=r_{p}$		

The entire SeO_4_^2–^ uptake process is described as a combination of macroscale (i.e., in bulk phase solution) and microscale (i.e., inside the ion-exchange resin) mass transfer processes. Macroscale mass accumulation for SeO_4_^2–^ (eq 15) is based on the concentration of SeO_4_^2–^ in bulk phase (*C*_*b*,SeO_4_^2–^_), which would transfer through the liquid film with a mass transfer coefficient of *k*_*m*_ with concentration gradient at the interphase. The bulk phase concentration of Cl^–^ (*C*_*b*,Cl^–^_) can be easily calculated because the total equivalent concentration in the solution is fixed due to electron balance (eq 16). Mass transfer (eq 17) in the resin phase is dependent on the pore space concentration (*C*_*p*,SeO_4_^2–^_), mass gain through ion exchange reaction (*q*_SeO_4_^2–^_), particle porosity (ε_*p*_), particle density (ρ_*p*_), and intraparticle pore diffusivity of SeO_4_^2–^ (*D*_*p*_). Specifically, the sorption rate was estimated based on the forward rate constant *k*_*f*_ and reverse rate constant *k*_*r*_ (eq 18). We need to note that *k*_*f*_ is expected to be large because ion exchange reactions usually proceed instantaneously. Given that the total ion exchange capacity (*q*_*T*_), which was determined from the fixed-bed column test using SeO_4_^2–^ and SO_4_^2–^ in this study, is fixed, the resin phase Cl^–^ concentration (*q*_Cl^–^_) can be calculated following the electron balance (eq 19). The average capacity of the resin $\left(\overset{®}{q_{{\mathrm{SeO}}_{4}^{2-}}\;}\right)$ is calculated based on eq 20. The initial and boundary conditions are expressed by eqs 21–23. During the simulations, parameters including *k*_*m*_, *D*_*p*_, *k*_*f*_, and reaction equilibrium constant *K* were optimized by minimizing an objective function, similar to that used in the equilibrium modeling (eq 12). The differential equations were solved using the Catalysis and Treatment System (CATS) modeling framework, which is built on top of the Multiphysics Object Oriented Simulation Environment (MOOSE) modeling framework.^9^

## 3 Results and Discussion

### 3.1 Morphology of IRA-900 from SEM Analysis

Figure 2 presents SEM images of the pristine IRA-900 collected in SEI mode on three different scales. This resin was composed of spherical beads with diameters ranging from 400 to 800 μm (Figure 2). The spherical shape of the beads is important for efficient ion exchange as it ensures uniform packing in fixed-bed columns and minimizes the formation of preferential flow channels. A low degree of aggregation that was visually observed in batch experiments where the resin was dispersed in solution also ensures a stable performance.^43^ At a higher magnification [Figure S1­(a), 30 μm scale], the surface of IRA-900 is smooth but slightly textured. At the highest magnification [Figure S1­(b), 10 μm scale], the surface has a rough, irregular texture with uneven features. Such surface characteristics may potentially contribute to increased surface area, enhancing interactions between the resin surface and ions in the solution. In addition, despite macroporous structure not being directly observable in Figure S1, previous studies have reported that the AmberLite IRA-900 resin has a smooth surface with numerous macroporous channels, formed during its polymerization process, which facilitate efficient internal mass transfer.^44,45^ Furthermore, macroporous resin beads are generally resistant to swelling, oxidation, and temperature changes. Although moderate shrinkage or swelling might occur during hydration or drying, fresh IRA-900 beads typically maintain structural integrity (spherical shape and intact surface) under moderate mechanical handling conditions.^45^ In addition, the beads’ robust sphere morphology is also frequently noted as a practical benefit, allowing easy separation and reuse.^44^

**Figure 2:**
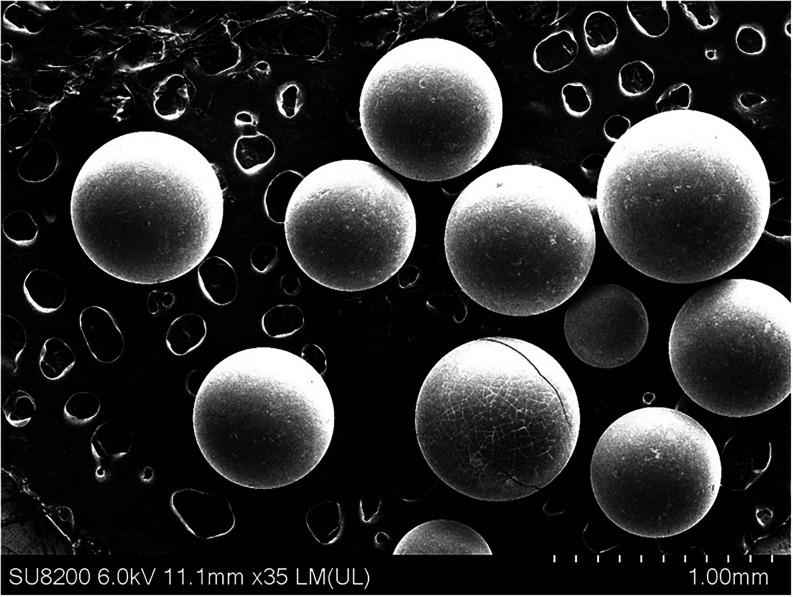
SEM images of pristine IRA-900 resin collected in SEI mode at 1 mm scale magnification showing uniform spherical beads (400–800 μm).

### 3.2 Effect of pH on Sorption of SeO_4_^2–^ and SeO_3_^2–^ by IRA-900

Figure 3 shows the effect of pH on the removal efficiency. Here, the reported pH values represent the final pH measured at equilibrium, as the equilibrium conditions ultimately govern the sorption behavior, while the initial solution pH values were adjusted to 2, 4, 6, 8, and 10. These results indicate an increase of SeO_3_^2–^ uptake with a rising pH. The pH-dependent sorption behavior found in this study can be explained by the speciation of Se (IV) under various pH values.^46^ From pH 2 to 4, both H_2_SeO_3_ and HSeO_3_^–^ coexist in solution, with the proportion of HSeO_3_^–^ increasing as pH rises. This trend aligns with the results shown in Figure 3, where the removal efficiency of Se (IV) remained relatively low but increased with rising pH, reflecting the speciation shift from H_2_SeO_3_ to HSeO_3_^–^. Between pH 4 and 6, the removal efficiency remains constant as the speciation of Se (IV) remains predominantly in the HSeO_3_^–^ form within this range. From pH 6 to 10, the removal efficiency increases, driven by the increasing fraction of SeO_3_^2–^ compared to HSeO_3_^–^. As such, although only total Se uptake was directly measured, the combined speciation distribution^46^ and removal trends indicated by Figure 3 allow inference of the selectivity order among the Se (IV) species. Specifically, this behavior implies that the sorption selectivity of IRA-900 follows the order, SeO_3_^2–^ > HSeO_3_^–^ > H_2_SeO_3_, indicating a stronger affinity for SeO_3_^2–^ over other Se (IV) species with different protonation states. Furthermore, Table S1 presents a comparison between the initial and equilibrium pH values. A noticeable decrease in pH was observed for the solutions initially adjusted to pH 8 and 10, where the equilibrium pH dropped to approximately 6 and 8, respectively, while only minor variations occurred between pH 2 and 6. Since the fraction of SeO_3_^2–^ continues to increase from pH 6 to 10,^46^ these pH changes do not affect the overall interpretation of the ion-exchange behavior based on speciation.

**Figure 3:**
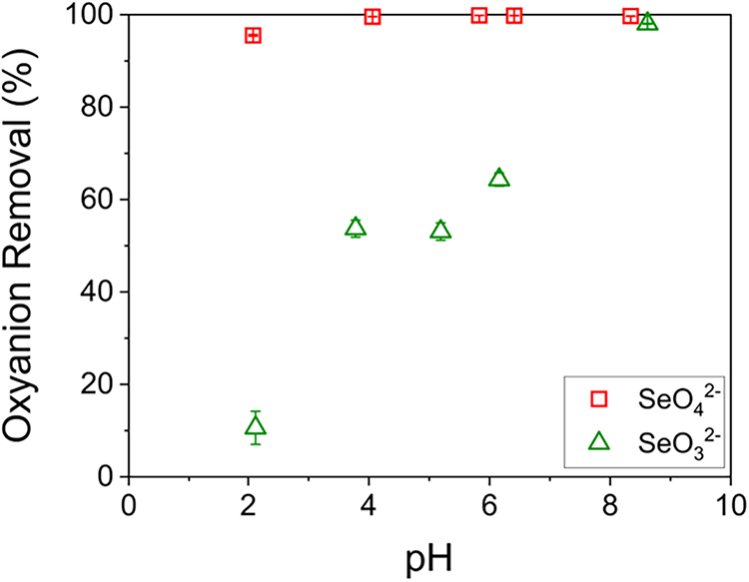
Sorption of Se by IRA-900 as a function of equilibrium pH using 20 g/L of IRA-900 and initial SeO_4_^2–^/SeO_3_^2–^ concentration of 1.5 mM in a 100 mL solution.

For SeO_4_^2–^, pH has a minimal effect on the removal efficiency within the tested pH range, as Se (VI) remains a fully deprotonated divalent anion between pH 4 to 14.^46^ The slight decrease in sorption observed around pH 2, similarly, can be explained by the reduced fraction of SeO_4_^2–^ and the increased fraction of HSeO_4_^–^, revealing a higher selectivity for SeO_4_^2–^ over HSeO_4_^–^. The higher SeO_3_^2–^ and SeO_4_^2–^ removal efficiencies than those for HSeO_3_^–^ and HSeO_4_^–^, respectively, suggest that IRA-900 interacts more strongly with fully deprotonated Se oxyanions through its functional groups. This trend suggests a predominance of electrostatic attraction in ion exchange and a low sorption tendency of protonated species. This difference is expected for charge-driven ion exchange processes. Due to the high pH dependency of SeO_3_^2–^ removal, optimizing the solution pH is expected to greatly improve the removal efficiency of Se in a treatment process. Moreover, the stability of SeO_4_^2–^ sorption across a broad pH range suggests that its removal efficiency is less sensitive to pH variations and thus more predictable in complex aqueous systems.

For clarity and consistency, Se (IV) is represented as SeO_3_^2–^ throughout this paper in both the experimental results and the modeling sections, even though different protonation states may dominate at specific pH values.

### 3.3 Batch Equilibrium Experimental Data

Batch equilibrium experiments were performed to obtain sorption isotherm data. IRA-900 was used for the removal of all three oxyanions SeO_4_^2–^, SeO_3_^2–^, and SO_4_^2–^ from single component aqueous solutions, and the sorption isotherms are shown in Figure 4. The results indicate that IRA-900 exhibits the highest sorption capacity (*q*_*e*_) for SeO_4_^2–^, followed by SO_4_^2–^ and SeO_3_^2–^, with the capacities following the order: SeO_4_^2–^ > SO_4_^2–^ > SeO_3_^2–^.

**Figure 4:**
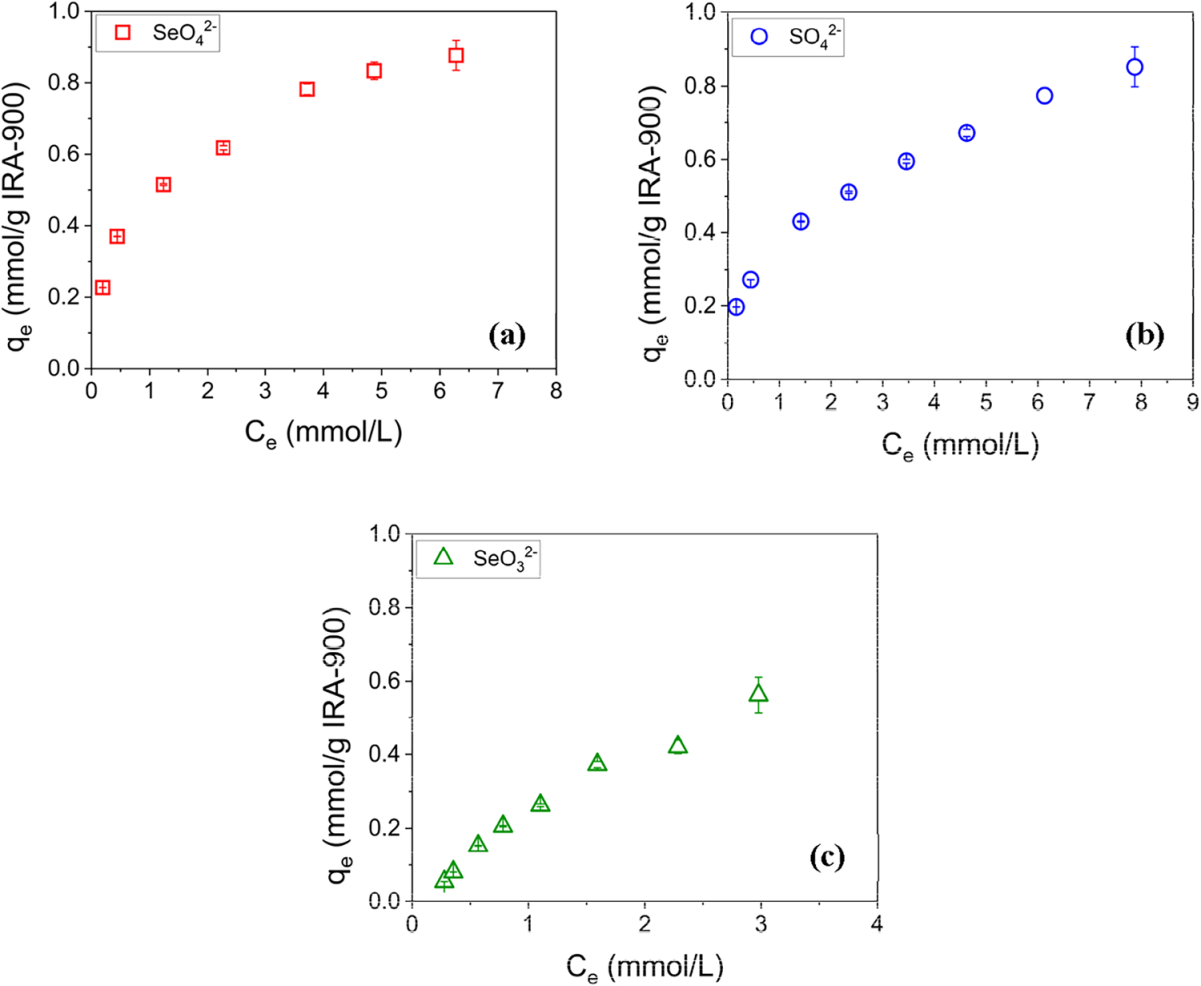
Sorption isotherms for (a) SeO_4_^2–^, (b) SO_4_^2–^, and (c) SeO_3_^2–^ for IRA-900. (*q*_*e*_: resin phase equilibrium sorption capacity; *C*_*e*_: equilibrium concentration in bulk solution).

Equilibrium data obtained from these experiments were used to estimate important sorption parameters. Such parameters are to be used in numerical models as a means to describe the sorption performance of IRA-900 under various conditions. In addition, the experimental data were benchmarked against simulation results as a means to check model validity.

In real SO_4_^2–^-rich industrial aqueous solutions, particularly when S concentrations greatly exceed those of Se, SO_4_^2–^ competes strongly with Se oxyanions during sorption or precipitation processes.^11^ It has been widely reported that SeO_4_^2–^ and SO_4_^2–^ share structural and chemical similarities, leading to similar sorption and interaction behaviors with multiple materials.^16–18,47^ Chubar and Szlachta examined the removal of SeO_3_^2–^ and SeO_4_^2–^ and found that SO_4_^2–^ had a significant impact on SeO_4_^2–^ removal using LDH-based sorbents, but not on SeO_3_^2–^ removal under various conditions, which suggests that SO_4_^2–^ competes strongly with SeO_4_^2–^, but not with SeO_3_^2–^.^47^

Equilibrium experiments for multicomponent solutions (e.g., binary solutions) were conducted to analyze the selectivity of IRA-900 for SO_4_^2–^ and SeO_4_^2–^/SeO_3_^2–^. Figure 5 shows that IRA-900 removes a higher fraction of SeO_4_^2–^ present in the solution than a fraction of SO_4_^2–^, even when SO_4_^2–^ is present in large excess. At the same time, however, the fraction of SO_4_^2–^ removed is still high, which implies strong competition with SeO_4_^2–^. Although both ions exhibited high removal efficiencies, which might be due to the excess resin mass, under resin-limited conditions SO_4_^2–^ would likely reduce SeO_4_^2–^ uptake. These results also indicate that, despite their identical charges and similar molecular geometries, SeO_4_^2–^ interacts more strongly with IRA-900 than SO_4_^2–^. In contrast, SeO_3_^2–^ sorption was significantly less than that of SeO_4_^2–^ or SO_4_^2–^ under the same conditions, which is consistent with previous studies.^48^ These observations can be attributed to the physicochemical properties of the ions, which are discussed in detail in Section 3.5.1.

**Figure 5:**
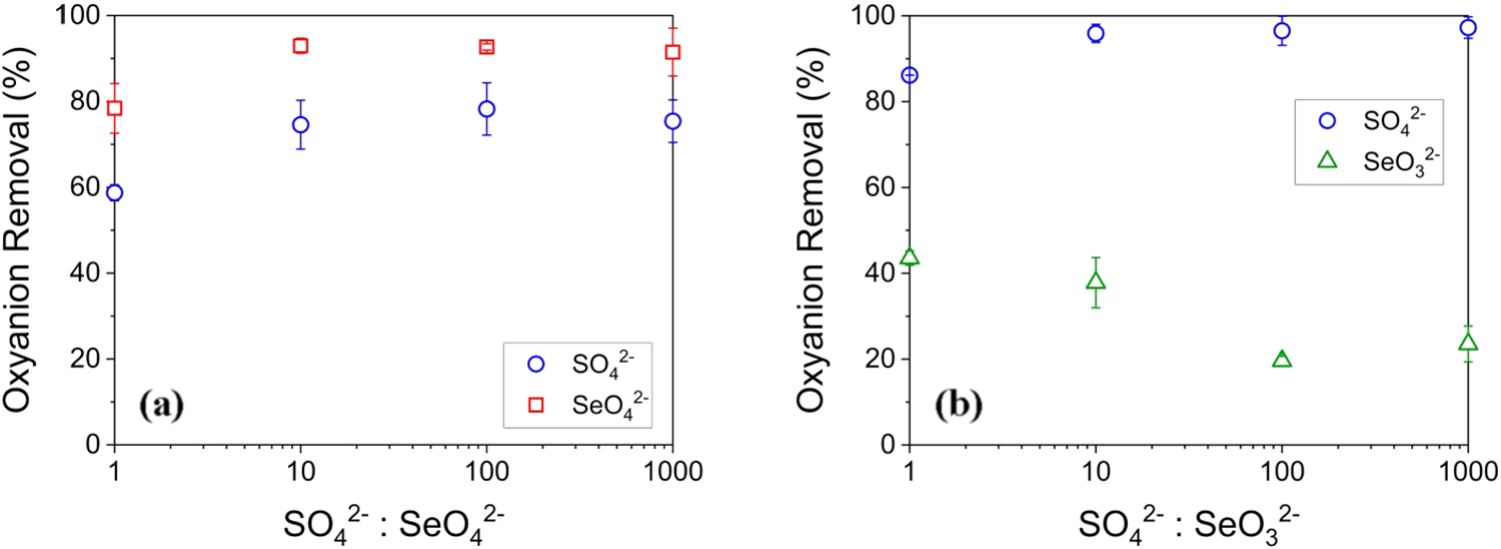
Oxyanion removal fraction at equilibrium using 20 g/L IRA-900 in binary solutions. The initial concentration of SO_4_^2–^ is fixed at 2.5 mM, while the initial concentrations of (a) SeO_4_^2–^ and (b) SeO_3_^2–^ decrease from 2.5 mM (1:1) to 0.0025 mM (1000:1).

### 3.4 Batch Kinetic Experiments

Kinetic data can be used to guide the design of unit operations, including batch and fixed-bed column systems, thus facilitating effective Se removal in actual treatment processes. They are helpful in determining appropriate contact times to optimize the removal efficiency and reduce operating costs, which are important for process evaluation. Kinetic data can be used to estimate model parameters accounting for the rates and mechanisms of Se and S removal.

Figure 6 includes kinetic data for the removal of SeO_4_^2–^, SO_4_^2–^, and SeO_3_^2–^ by the IRA-900 resin. Equilibrium was achieved relatively rapidly, within approximately 120 min for SeO_4_^2–^ and SO_4_^2–^, and around 150 min for SeO_3_^2–^, highlighting the comparatively fast kinetics of the ion-exchange process. Additionally, the equilibrium time exhibited minimal variation across different initial concentrations (1, 5, 10, and 20 mM), indicating that the initial ion concentration did not significantly affect the time to reach equilibrium under these experimental conditions. Furthermore, the figures also indicate the differences in time to reach equilibrium among the three ions. SeO_3_^2–^ always takes longer to reach equilibrium than either SeO_4_^2–^ or SO_4_^2–^, which may be attributed to its lower affinity for the resin that was determined in Section 3.3. Such reduced affinity potentially decreases the sorption driving force, resulting in slower uptake kinetics. These findings highlighted the need for further examination of transport mechanisms to better interpret the kinetic behavior discussed in Section 3.5.2.

**Figure 6:**
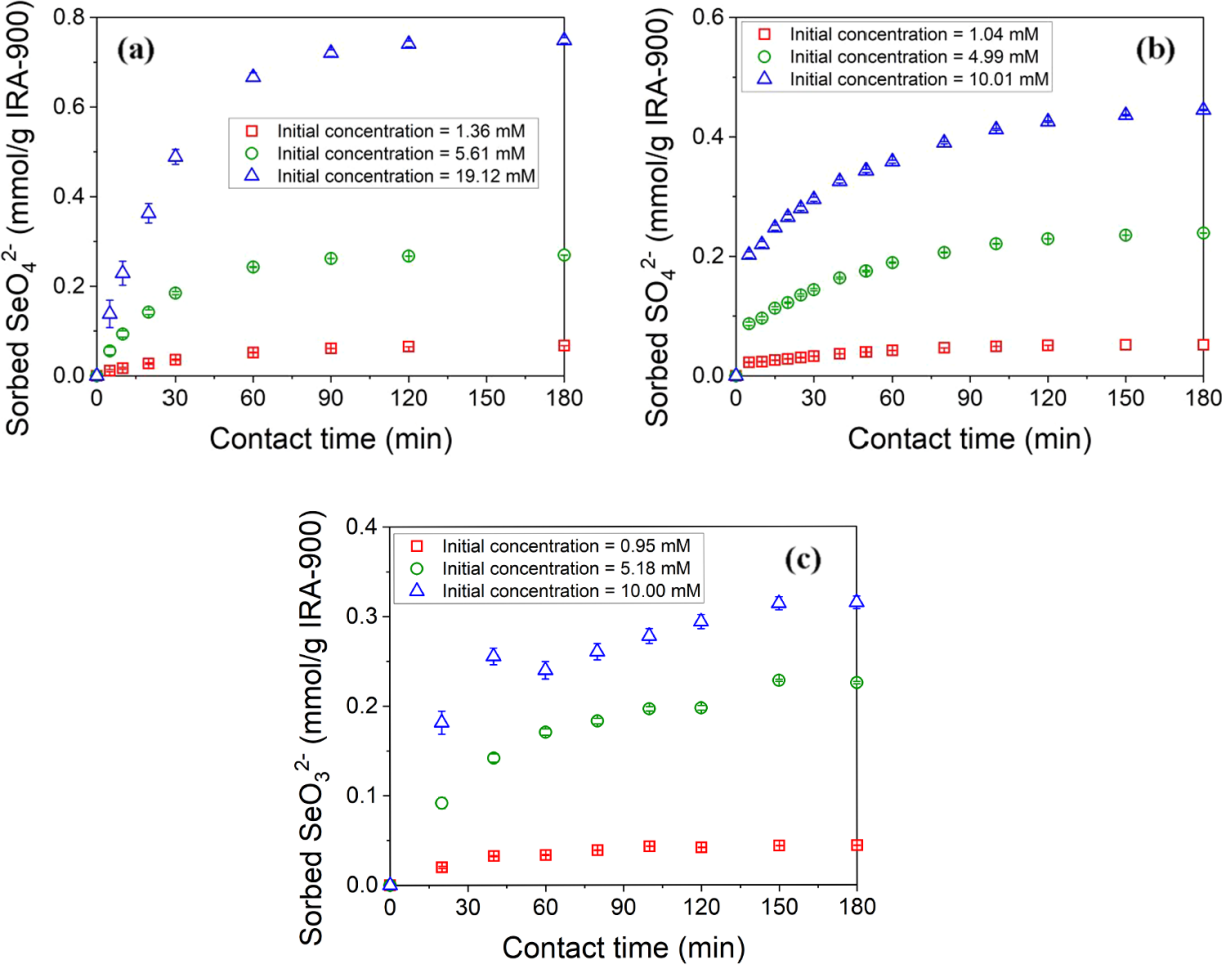
Kinetics of uptake by IRA-900 for (a) SeO_4_^2–^ at 1 mM, 5 mM, and 20 mM; (b) SO_4_^2–^ at 1 mM, 5 mM, and 10 mM; and (c) SeO_3_^2–^ at 1 mM, 5 mM, and 10 mM initial concentrations. Resin phase sorption capacity (*q*, mmol/g) is shown as a function of contact time (*t*, min) using a resin dosage of 20 g/L in 100 mL solutions for 180 min.

### 3.5 Modeling Results

#### 3.5.1 Equilibrium

Experimental data obtained from batch equilibrium experiments were used for model parameter estimation. The total exchange capacity was determined to be 2.04 mequiv/g from fixed-bed experiments using SeO_4_^2–^ and SO_4_^2–^ (Table S2). The optimized LMA model parameters in Cl^–^/SeO_4_^2–^, Cl^–^/SO_4_^2–^, and Cl^–^/SeO_3_^2–^ systems are listed in Table S3. The experimental data and simulated equivalent fraction isotherms are shown in Figure 7. As shown in Figure 7, the *R* ^2^ values for the curves are larger than 0.9, indicating that the simulated equilibrium isotherms agree well with experimental observations. Specifically, both ideal and nonideal models accurately describe the sorption behavior of three oxyanions. The utilization of nonideal models, however, does not result in significant improvement in accuracy compared to ideal models under the present experimental conditions, and the obtained *K*_*A*_^*B*^ and *k*_*A*_^*B*^ values are similar. This is primarily because the ionic strength of the solution was very low (<0.005 M, estimated from the initial concentrations of the oxyanions in the prepared solutions), which caused the activity coefficients to be close to 1 and thus minimized deviations from ideal behavior. It should also be noted that the nonideal simulation of SO_4_^2–^ and SeO_3_^2–^ exhibited potential overfitting trends despite their high R^2^ values, which may be attributed to the additional adjustable parameters introduced in the nonideal model. This reflects a limitation of applying a more complex formulation to low-ionic-strength systems and underscores the practical value of the ideal LMA approach: It provides reliable equilibrium descriptions with explicit physicochemical meaning while avoiding unnecessary complexity in low-ionic-strength systems. The nonideal model was, however, retained in this work for completeness and for potential application to higher-ionic-strength conditions where activity corrections become more significant. Overall, the LMA model effectively describes the ion-exchange equilibrium of SeO_4_^2–^, SO_4_^2–^, and SeO_3_^2–^ on the IRA-900 resin at neutral pH.

**Figure 7:**
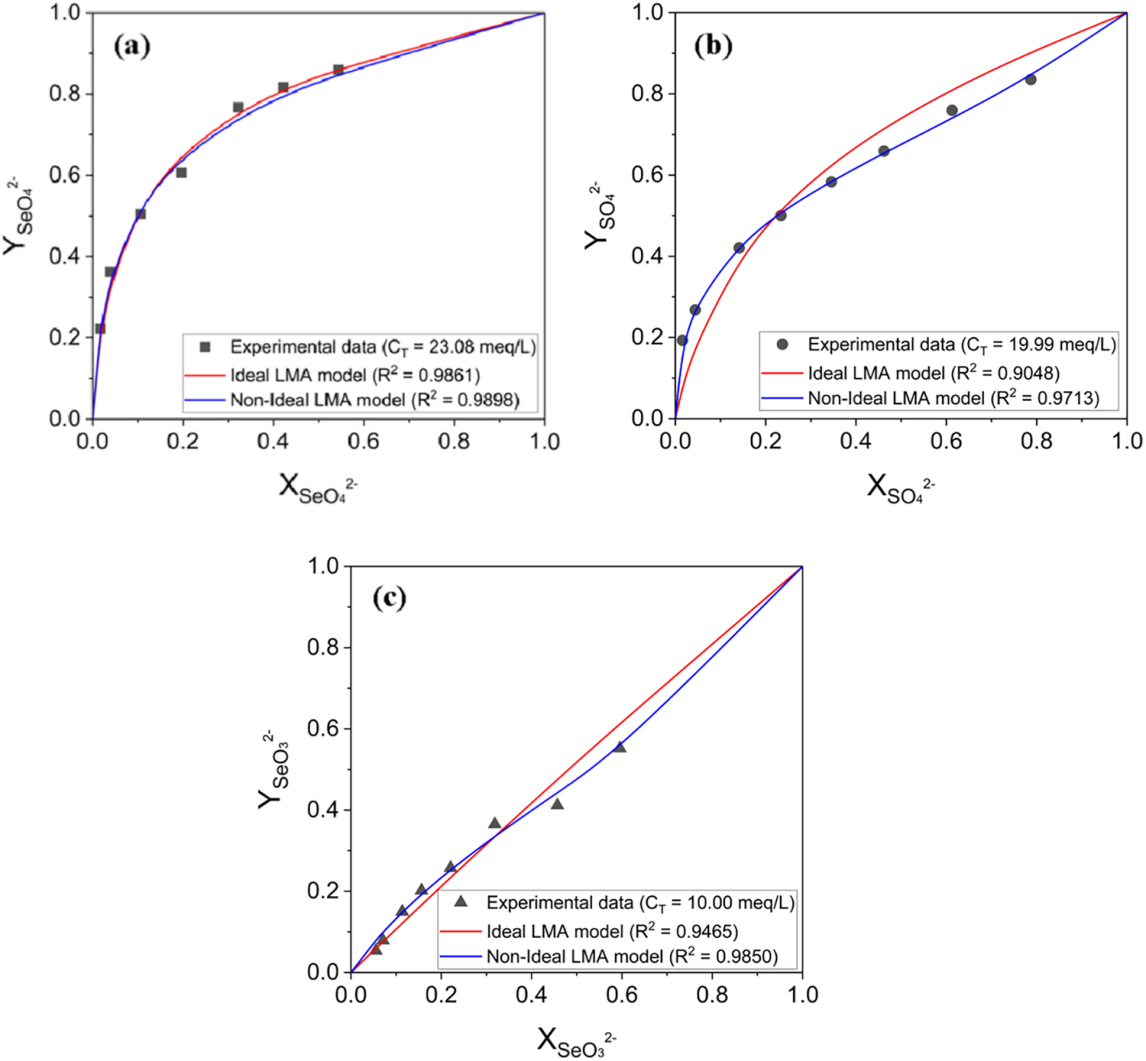
Experimental and simulated equilibrium isotherms in terms of equivalent fractions for (a) SeO_4_^2–^, (b) SO_4_^2–^, and (c) SeO_3_^2–^ solutions at different initial concentrations. The horizontal and vertical axes represent the equivalent fractions of oxyanions in the solution phase and resin phase, respectively. The black dots are experimental data obtained from batch equilibrium experiments, and the solid lines are simulated isotherms using LMA models.

The separation factor, defined by eq 13, is a metric that reflects the relative selectivity of two species. For instance, the separation factor for SO_4_^2–^ relative to SeO_4_^2–^ (α_SeO_4_^2–^_^SO_4_^2–^^), calculated using eq 14, is 0.27. This value, being less than 1, suggests that IRA-900 has a lower sorption affinity for SO_4_^2–^ than SeO_4_^2–^. To examine the separation factors more intuitively, the theoretical curves for α_*B*_^*A*^ = 10, 1, 0.1 are plotted in Figure 8 along with the experimental equilibrium isotherms. The separation factors for SeO_4_^2–^ and SO_4_^2–^ yielded values greater than 1, suggesting these oxyanions exhibit higher affinity over Cl^–^. Conversely, SeO_3_^2–^ exhibits the lowest separation factor, approaching a value close to 1, which is nearly comparable to that of Cl^–^, indicating lowest affinity. These findings are consistent with the experimental data of Figure 5.

**Figure 8:**
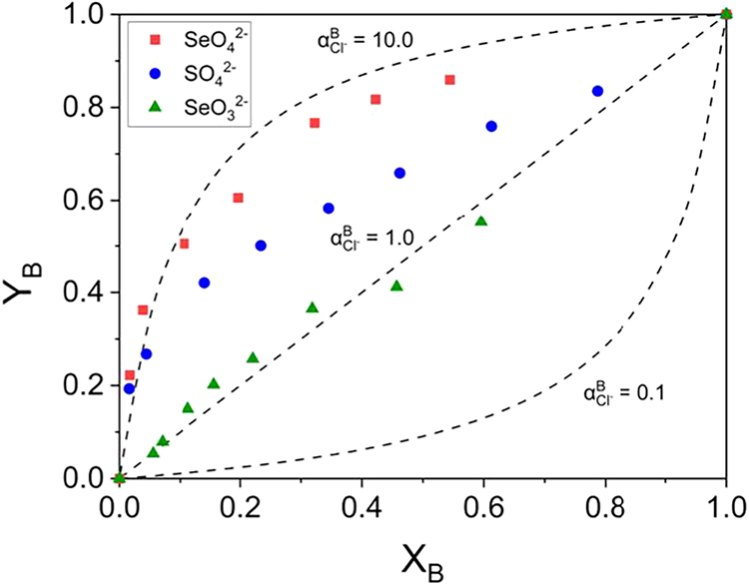
Equilibrium isotherms illustrating the separation factors (α_*B*_^*A*^) for SeO_4_^2–^, SO_4_^2–^, and SeO_3_^2–^ relative to Cl^–^. The dashed lines represent theoretical curves corresponding to separation factor values (α_*B*_^*A*^) of 10, 1, and 0.1.

To rationalize the consistently lower affinity of SeO_3_^2–^ relative to both SeO_4_^2–^ and SO_4_^2–^, multiple contributing factors must be considered. The dominant influence is its speciation at near-neutral pH, where more than 80% of Se (IV) exists as the monovalent HSeO_3_^–^,^46^ which exhibits much lower exchange affinity than divalent oxyanions. Strong base anion resins intrinsically favor divalent ions through stronger electrostatic interactions and the bisite pairing requirement at closely spaced quaternary ammonium groups.^49–51^ Even when present as SeO_3_^2–^, it exhibits the largest hydrated radius among the oxyanions (4.36 Å versus 3.94 Å for SeO_4_^2–^ and 3.79 Å for SO_4_^2–^).^52–54^ A larger hydrated radius lowers the effective charge density and weakens electrostatic interactions with the fixed quaternary ammonium sites of the resin. Consequently, the more compact SeO_4_^2–^ and SO_4_^2–^ bind more strongly than SeO_3_^2–^.^55,56^ In addition, molecular geometry further modulates resin affinity. SeO_4_^2–^ is symmetric and tetrahedral, similar to SO_4_^2–^, whereas SeO_3_^2–^ is asymmetric and trigonal pyramidal due to a lone electron pair.^53^ This asymmetry concentrates negative charge on only three oxygens, produces an anisotropic hydration shell, and restricts favorable orientations for electrostatic binding. Together, the unfavorable speciation, enlarged hydrated radius, reduced charge density, and potential geometric constraints explain the consistently weaker preference for Se (IV) compared with SeO_4_^2–^ and SO_4_^2–^ in the ion-exchange process.

Although SeO_4_^2–^ and SO_4_^2–^ are both divalent and share a symmetric tetrahedral geometry,^53^ their affinities for IRA-900 differ systematically. SO_4_^2–^ has a more exothermic hydration enthalpy (−1035 kJ mol^–1^) than SeO_4_^2–^ (−900 kJ mol^–1^),^57,58^ indicating stronger hydration. According to ion-exchange theory, more strongly hydrated ions have lower affinity for the resin.^59^ Consequently, the less strongly hydrated SeO_4_^2–^ is exchanged more readily than SO_4_^2–^. This trend also reflects charge density considerations: SO_4_^2–^ concentrates its −2 charge over a smaller ionic framework, producing higher charge density and therefore stronger electrostatic interactions with surrounding water molecules, whereas SeO_4_^2–^ distributes the same charge more diffusely, resulting in weaker electrostatic interactions.^55,56^ Furthermore, the higher affinity of SeO_4_^2–^ compared to that of SO_4_^2–^ can potentially be explained by its greater polarizability, which is recognized as an important determinant of ion-exchange selectivity.^60^ Taken together, the combination of weaker hydration enthalpy, lower charge density, and enhanced polarizability explain why SeO_4_^2–^ is consistently favored over SO_4_^2–^ despite their comparable size and geometry.

For Cl^–^ ions, which initially occupy the resin phase and are exchanged with target oxyanions, it is important to consider their intrinsic affinity. Although the hydrated radius of Cl^–^ (∼3.2 Å) is smaller than those of SeO_4_^2–^ (3.94 Å), SO_4_^2–^ (3.79 Å), and SeO_3_^2–^ (4.36 Å),^52–54,61^ its monovalent charge fundamentally limits its interaction with the resin sites.^49,50^ Cl^–^ also has a lower effective charge density than the oxyanions, resulting in weaker electrostatic stabilization at the quaternary ammonium sites despite its relatively small hydrated size.^55,56^ Additionally, its low polarizability results in intrinsically weak affinity.^60^ Modeling results further show that Se (IV) and Cl^–^ exhibit nearly identical affinity (α_Cl^–^_^SeO_3_^2–^^ ≈ 1), which reflects the predominance of HSeO_3_^–^ at near-neutral pH. As both species are monovalent, their behavior is governed primarily by valence under comparable conditions.

All relevant physicochemical factors including speciation, hydration, charge density, and polarizability together govern the observed selectivity, making Se (IV) the species with the weakest affinity relative to SeO_4_^2–^ and SO_4_^2–^. Cl^–^, although similar in size, also shows weak affinity due to its single charge and low charge density. The resulting selectivity order is SeO_4_^2–^ > SO_4_^2–^ > SeO_3_^2–^ ≈ Cl^–^, consistent with equilibrium experimental and modeling results.

#### 3.5.2 Kinetics

The optimized kinetic parameters and corresponding *R* ^2^ values for each set of experiments are summarized in Table S4. Simulated results are plotted in Figure 9 along with experimental data for comparison. The parameters obtained provided a good description (i.e., *R* ^2^ > 0.9 for all cases) for both the average resin phase concentration [Figure 9(a,c,e)] and the bulk phase concentration [Figure 9(b,d,f)].

**Figure 9:**
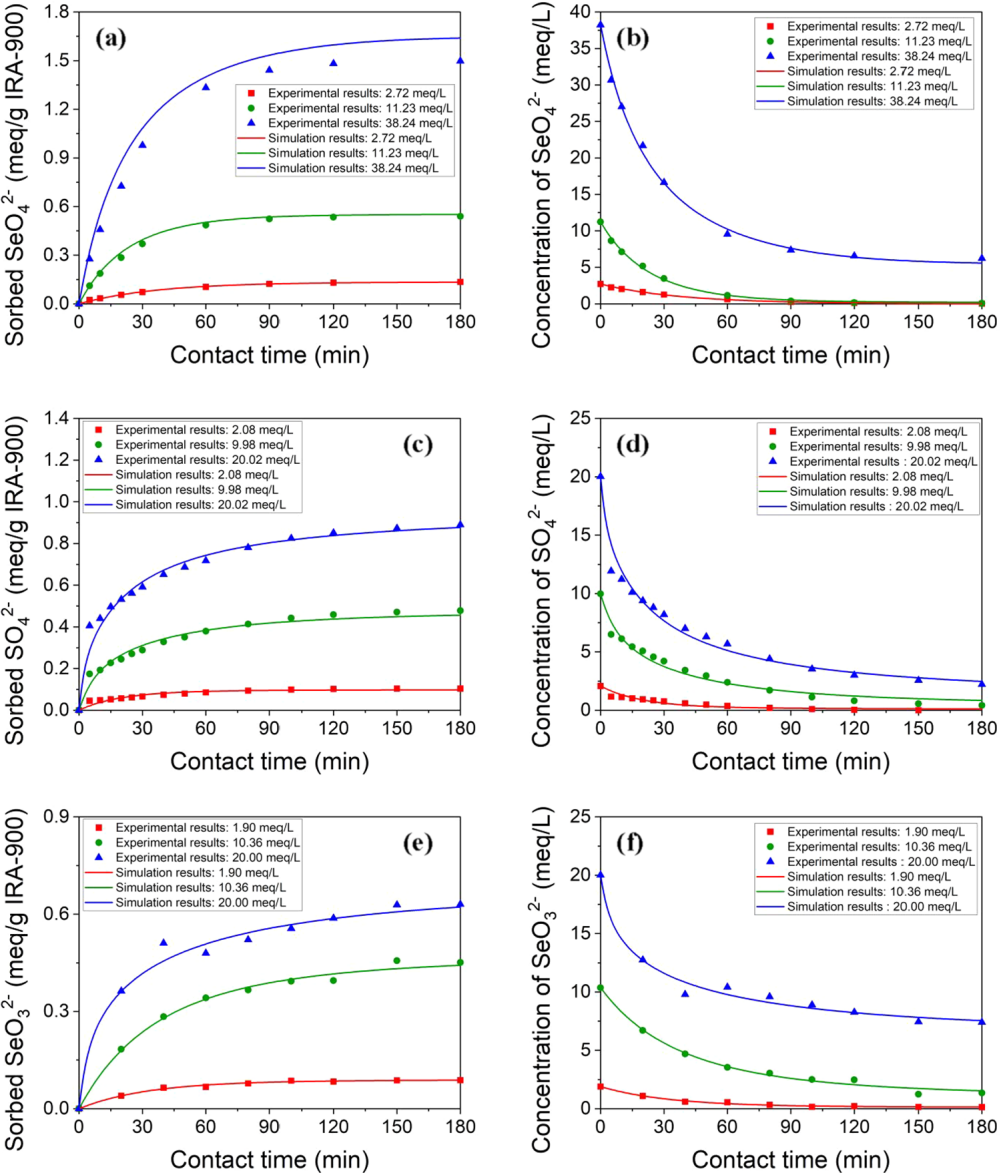
Plots of ion-exchange kinetics for the capacities of (a) SeO_4_^2–^, (c) SO_4_^2–^, and (e) SeO_3_^2–^ in the resin phase (*q*), and concentrations of (b) SeO_4_^2–^, (d) SO_4_^2–^, and (f) SeO_3_^2–^ in the bulk solution (*C*_*b*_). Dots represent experimental data and lines represent simulated results.

The equilibrium reaction constant (*K*), defined as the ratio of forward and reverse reaction rates, follows the order SeO_4_^2–^ > SO_4_^2–^ > SeO_3_^2–^ (Table S4). This finding aligns with both the LMA equilibrium modeling and the experimental observations. The forward rate constants for the oxyanions uptake are also much higher than the corresponding backward rate constants for desorption, similar to findings reported for nitrate sorption on AmberLite IRA-400.^42^

The film mass-transfer coefficient (*k*_*m*_) was highest for SO_4_^2–^, followed by SeO_4_^2–^ and SeO_3_^2–^ (Table S4). Although the film mass-transfer coefficient is primarily governed by external boundary-layer and hydrodynamic conditions, intrinsic molecular properties such as hydrated-ion sizes and diffusivities indirectly affect these values. Ions with higher intrinsic diffusivities generally exhibit higher film transfer rates when film thickness remains constant, as outlined by the Nernst film theory.^55^ In this study, SO_4_^2–^ has the smallest hydrated radius (3.79 Å) compared with SeO_4_^2–^ (3.94 Å) and SeO_3_^2–^ (4.36 Å) and correspondingly exhibits the highest film mass-transfer coefficient,^52–54^ which agrees with the established understanding that ions of smaller hydrodynamic radius typically diffuse more rapidly across external films. The significant increase in the film mass-transfer coefficient of SO_4_^2–^ with concentration in Table S4 further reflects this behavior. The influence of hydrated size on mass transfer has also been demonstrated for halogen ions, where the transfer rates increase in the order F^–^ < Cl^–^ < Br^–^ < I^–^, which inversely correlates with their hydrated sizes.^62^

Intraparticle (pore) diffusivities (*D*_*p*_) reflect the influence of ion size, hydration, and binding strength on the ease with which ions migrate through the water–polymer network of the resin. The values of pore diffusivity show a clear trend: SeO_4_^2–^ exhibit the highest diffusivity, whereas SO_4_^2–^ and SeO_3_^2–^ display similar but lower values (Table S4). This behavior can primarily be attributed to differences in hydration energy, hydrated-ion radius, and resin-ion interactions. SeO_4_^2–^ has a lower hydration energy (∼900 kJ/mol) than SO_4_^2–^ (∼1080 kJ/mol),^57^ which allows it to more readily exchange its hydration shell when entering the resin phase, facilitating faster transport within the polymer network. The limited diffusivity of SeO_3_^2–^ is comparable to SO_4_^2–^ partly due to its weaker or less favorable interactions with resin functional groups and its presence in monovalent form as HSeO_3_^–^, which displays distinct transport behavior. Overall, the results suggest that SO_4_^2–^ has the highest film mass-transfer coefficient, whereas SeO_4_^2–^ has the highest pore diffusivity among the three oxyanions.

When examining the effect of initial concentration, the equilibrium constant first increases and then decreases as the initial concentration rises (Table S4), which may be related to changes in solution ionic strength. Prior studies report that increasing ionic strength can reduce anion sorption affinity; for example, the sorption capacity for certain anions can decrease by up to 50% when the ionic strength of a background electrolyte reaches approximately 0.3–0.5 M.^63^ At high ionic strength, compression of the electrical double layer weakens electrostatic attraction, and counterions accumulate around sorption sites, partially neutralizing the surface charge and further reducing the electrostatic interaction between sorption sites and the sorbent.^63^

The external film mass-transfer coefficient (*k*_*m*_) is primarily governed by hydrodynamics and is often assumed to remain constant for a given system. A recent study reported film diffusion coefficients between 0.0052 and 0.0071 cm/s across various nitrate concentrations, demonstrating that initial concentration and resin dosages can influence film mass-transfer.^64^ In this study, higher initial concentrations similarly produced higher *k*_*m*_ (Table S4). A plausible explanation is that a steeper concentration gradient at higher solute levels increases the driving force for transport, enhancing film mass transfer through ion–ion electrostatic interactions. In multicomponent systems, the presence of additional ions of other species elevates the overall ionic strength, which can modify the physicochemical properties of the boundary film and reduce the effective external mass-transfer coefficient.^65,66^

In this study, the pore diffusion coefficients varied with different initial ion concentrations (Table S4). Both SeO_4_^2–^ and SO_4_^2–^ showed increasing pore diffusivity as their initial concentrations increased, whereas SeO_3_^2–^ displayed an opposite trend [Table S4­(a,b)]. Similar variability and nonmonotonic behavior of pore diffusivity with changing initial concentration have been reported for other anions, such as nitrate.^64^ Previous research has also demonstrated that the internal morphology of ion-exchange resins significantly affects pore diffusivity. In macroporous exchangers, increasing the concentration of competing Cl^–^ significantly enhances the effective diffusivity due to the interconnected pore network that promotes transport between microgels, while gel-type exchangers lacking such networks show negligible sensitivity of pore diffusivity to concentration changes.^67^ Although these studies typically involve competing ion scenarios, the underlying relationship between concentration gradients and intraparticle transport still applies to single-ion systems. In our system, higher initial concentrations likely increased intraparticle concentration gradients, influencing diffusivity for SeO_4_^2–^ and SO_4_^2–^. The opposing behavior observed for SeO_3_^2–^ may be attributed to its relatively low resin affinity arising from structural differences or the coexistence of multiple Se (IV) species.

### 3.6 Surface Analysis by XPS

XPS characterization was performed to compare the pristine IRA-900 with resins loaded from single-anion SeO_4_^2–^, single-anion SeO_3_^2–^, and equimolar SeO_4_^2–^/SeO_3_^2–^ solutions to investigate the key mechanism regarding the ion-exchange behavior between Cl^–^ and Se oxyanions. From XPS survey spectra (Figure 10), Se was observed only on the loaded resin samples and not on the pristine resin, consistent with Se uptake by IRA-900. Upon loading, inorganic Cl decreased markedly, consistent with its exchange with Se oxyanions. Quantitative atomic fractions from surface composition analysis in Table 4 further indicate that the atomic percentage (at.%) of inorganic Cl decreased from 3.45% on the pristine resin to 1.25, 0.6, and 0.75 at. % on the SeO_3_^2–^-loaded resin, SeO_4_^2–^-loaded resin, and mixture-loaded resin, respectively, while the total Se concurrently increased from zero (no Se detected) to 1.6, 2.65, and 2.2 at. % on the same resins, respectively. The total atomic concentration of Cl and Se kept nearly consistent, indicating the ion-exchange behavior between Cl^–^ and Se oxyanions.

**Figure 10:**
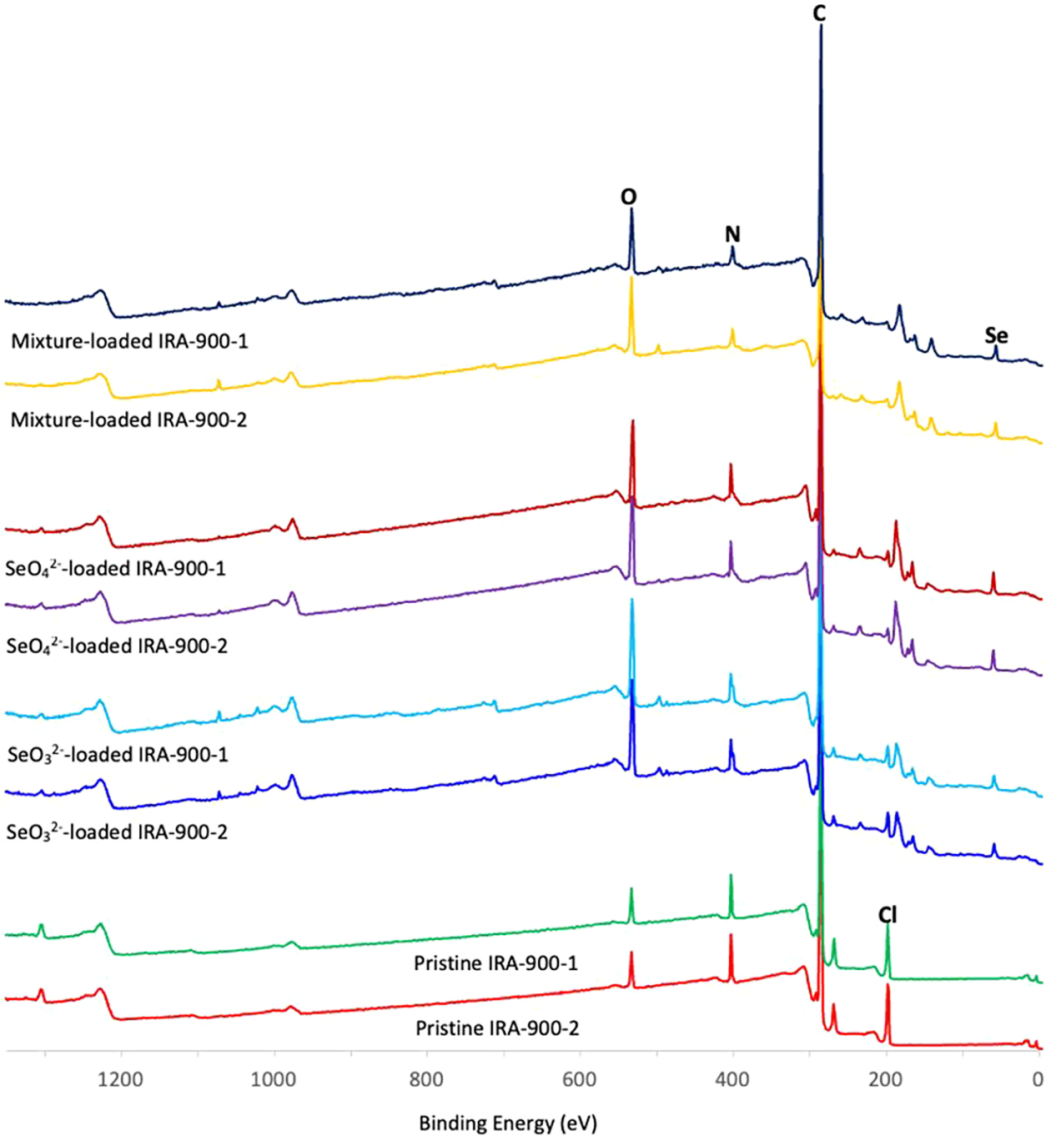
XPS survey spectra of IRA-900 (two replicates for each sample): pristine, SeO_4_^2–^-loaded, SeO_3_^2–^-loaded, and 1:1 SeO_4_^2–^/SeO_3_^2–^-loaded.

**Table 4:** Surface Elemental Composition of Pristine and Se-Loaded IRA-900 Resins from XPS Analysis^a^

resin	inorganic Cl	total Se
pristine IRA-900	3.45	0
SeO_3_^2–^-loaded IRA-900	1.25	1.60
SeO_4_^2–^-loaded IRA-900	0.60	2.65
mixture-loaded IRA-900	0.75	2.20

a All values are given in atomic percent (at.%).

High-resolution Se 3d spectra were used to determine the oxidation state of the sorbed Se species (Figure S2). The Se 3d peaks for the SeO_3_^2–^-loaded resin and SeO_4_^2–^-loaded resin appeared at 58–59 eV and 59–60 eV, corresponding to Se (IV) and Se (VI), respectively. While the peaks are close, they are observable and consistent with reported literature values.^21,68,69^ In the mixture-loaded resin, however, only a single Se 3d peak was observed, located between the peaks of Se (IV) and Se (VI), which can be explained by the overlap of Se (IV) and Se (VI) peaks, making it difficult to distinguish the individual species. From another perspective, the total atomic percentage of Se detected on the resins (Table 4) followed the expected order of uptake: SeO_4_^2–^-loaded (∼2.6 at. %) > mixture-loaded (∼2.1 at. %) > SeO_3_^2–^-loaded (∼1.5 at. %). This is consistent with the bulk equilibrium findings that IRA-900 has a higher capacity for SeO_4_^2–^ than for SeO_3_^2–^. Overall, the features observed from XPS align closely with the bulk phase in which the beads were prepared, with the SeO_4_^2–^-, SeO_3_^2–^-, and mixture-loaded resins showing results consistent with their respective solution oxidation states. Therefore, the XPS results demonstrate that the exchange behavior arises from ion exchange of Cl^–^ with Se oxyanions, with no evidence for reduction or oxidation under the conditions of this study.

## 4 Conclusions

This study systematically evaluated the performance of the strong-base anion-exchange resin IRA-900 for removing Se oxyanions (SeO_4_^2–^ and SeO_3_^2–^) and competing SO_4_^2–^ from aqueous solutions. Batch experiments demonstrated that IRA-900 exhibits high selectivity and substantial capacity for SeO_4_^2–^ even in the presence of competing SO_4_^2–^ at high relative concentrations, whereas the removal of SeO_3_^2–^ was considerably lower, reflecting the resin’s species-specific affinity and the complexity introduced by coexisting species of anions commonly encountered in industrial aqueous systems. The influence of pH on Se removal was also examined, indicating that pH control can contribute to optimizing treatment performance in systems containing SeO_3_^2–^. XPS analysis provided direct elemental-level evidence for the ion-exchange mechanism by confirming the displacement of Cl^–^ by Se oxyanions. Although distinguishing the oxidation state of sorbed Se was challenging, the overall Se uptake trends from XPS were consistent with our bulk equilibrium data, strengthening the mechanistic conclusions. Practical considerations confirm that IRA-900 offers a favorable balance of selectivity, capacity, spherical bead morphology suitable for packed-bed columns, and low cost and wide availability,^10^ making it an attractive option for treating industrial aqueous solutions.

Equilibrium data were accurately described by the LMA model, which effectively captured resin selectivity and competitive interactions. The derived parameters confirmed the preferential uptake order of SeO_4_^2–^ > SO_4_^2–^ > SeO_3_^2–^, aligning with experimental observations and providing strong basis for practical design and descriptive modeling of multicomponent ion-exchange systems. Kinetic analysis identified film and intraparticle diffusion processes as key contributors to the overall rate of Se removal. Numerical modeling incorporating these transport processes captured the experimental data, demonstrating its value as a descriptive tool for engineering design, optimization, and scale-up of ion-exchange treatment units.

While this study provides critical insights into the removal of Se oxyanions by IRA-900, certain limitations should be noted. The work relied primarily on batch equilibrium and kinetic experiments, which do not fully represent dynamic conditions of continuous-flow systems. Future studies must include breakthrough curve analysis in fixed-bed columns to validate the process performance under realistic operating conditions. In addition, although SO_4_^2–^ was selected as the primary competitor, real industrial waters contain other interfering ions such as carbonate species (HCO_3_^–^/CO_3_^2–^), nitrate (NO_3_^–^), and phosphate species (H_2_PO_4_^–^/HPO_4_^2–^). On an equivalent-charge basis, divalent carbonate (CO_3_^2–^) and phosphate (HPO_4_^2–^) are expected to compete more strongly with SeO_4_^2–^ than with monovalent NO_3_^–^, and their influence is pH dependent due to shifts in speciation. Future work aims to evaluate these competitive effects in ternary and quaternary systems, including real industrial matrices across practical pH ranges, to quantify their impact on SeO_4_^2–^ and SeO_3_^2–^ uptake. Furthermore, since all experiments were conducted at 20 °C and IRA-900 can operate across a broad temperature range (5–100 °C),^35^ additional studies should assess temperature effects in this large range to confirm its applicability under more variable environmental and industrial conditions.

Regarding practical deployment, regeneration and long-term stability of IRA-900 are critical considerations. While regeneration was outside the scope of this study, prior work by Tan et al. demonstrated effective regeneration using dilute HCl within 20 min. Although the resin performance decreased over 30 cycles, they identified an optimal reusability of approximately 6 cycles, noting that stronger acid regenerants could cause physical damage to the resin beads.^10^ These findings support the feasibility of resin reuse, but further work is needed to optimize regeneration protocols and evaluate long-term stability, particularly when treating real industrial water. The equilibrium and modeling results presented here also confirm that IRA-900 is highly effective for SeO_4_^2–^, but exhibits much lower affinity for SeO_3_^2–^. A potential solution is a two-stage treatment process that integrates IRA-900 with other materials more effective for SeO_3_^2–^, such as iron oxides (e.g., goethite, hematite, etc.), which have been proved in previous studies and in our preliminary results to be efficient and cost-effective for SeO_3_^2–^ capture.^19^ Addressing these critical factors is essential for establishing reliable Se treatment strategies.

Finally, while only single-component numerical models were presented here, the mechanistic framework discussed in this study can be extended to multicomponent systems in both batch and fixed-bed configurations. The framework is inherently flexible and can be expanded to incorporate additional parameters. Future developments will include temperature-dependent transport and reaction behavior as well as pH-driven speciation to create a more comprehensive model.

## Supplementary Material



## Notes

SEM images of pristine IRA-900 resin (Section S1), equilibrium pH measurements for SeO_4_^2–^ and SeO_3_^2–^ systems (Section S2), determination of the maximum ion-exchange capacity of IRA-900 based on fixed-bed saturation experiments (Section S3), optimized equilibrium and kinetic parameters (Section S4), high-resolution XPS spectra of the Cl 2p and Se 3d regions for pristine IRA-900 and oxyanion-loaded resins (Section S5) (PDF)
