# Supplementary material for: Removal of Selenium Oxyanions from Aqueous Solutions by Ion Exchange: Equilibrium, Kinetics, and Mechanistic Modeling
Source: ACS ES T Water. 2025 Dec 23;6(1):438–54. doi: 10.1021/acsestwater.5c01136 (PMC12797240; doi:10.1021/acsestwater.5c01136)
Supplement: Supplementary file 1 [file ew5c01136_si_001.pdf]

Removal of Selenium Oxyanions from Aqueous Solutions by Ion Exchange: Equilibrium, Kinetics, and Mechanistic Modeling

Zeng, Z.;[1] Shen, Z.;[1] Einkauf, J.D.;[2] Ladshaw, A.P.;[1] Custelcean, R.;[2] Tsouris, C.;[1,2] Yiacoumi, S.[1*]

[1]School of Civil and Environmental Engineering, Georgia Institute of Technology, Atlanta, GA, 30332

[2]Oak Ridge National Laboratory, Oak Ridge, TN, 37831

*Corresponding authors: sotira.yiacoumi@ce.gatech.edu

Notice of Copyright: This manuscript has been authored by UT-Battelle, LLC, under contract DE-AC05-00OR22725 with the U.S. Department of Energy (DOE). The U.S. government retains and the publisher, by accepting the article for publication, acknowledges that the U.S. government retains a nonexclusive, paid-up, irrevocable, worldwide license to publish or reproduce the published form of this manuscript, or allow others to do so, for U.S. government purposes. DOE will provide public access to these results of federally sponsored research in accordance with the DOE Public Access Plan (http://energy.gov/downloads/doe-public-access-plan).

Supplemental Information:

Section S1. Supplemental SEM Images of IRA-900 Resin.

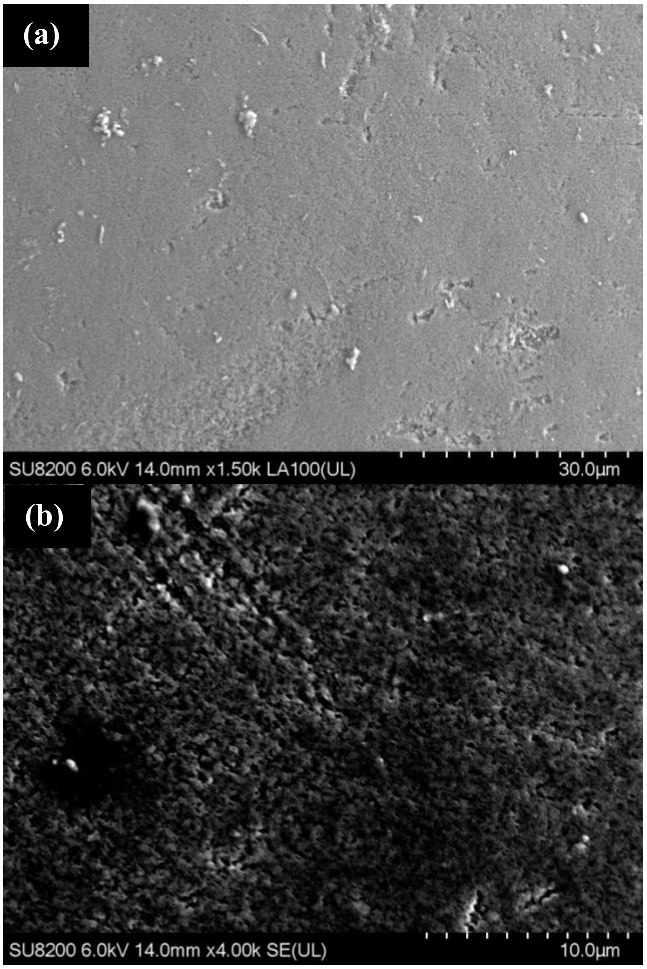

Figure S1. Supplemental SEM images of pristine IRA-900 resin collected in SEI mode: (a) 30-μm scale magnification showing a smooth but slightly textured surface; (b) 10-μm scale magnification showing a rough, irregular surface texture with uneven features.

Section S2. Final pH Measurements for SeO_4^{2-} and SeO_3^{2-} Equilibrium Experiments

Table S1. Comparison between initial and final (equilibrium) pH values during SeO_4^{2-} and SeO_3^{2-} ion-exchange experiments with IRA-900. Each experiment was performed using 20 g/L resin dosage in 100 mL of 1.5 mM solution at 20 °C for 24 h. Measured pH values were within ± 0.05 of the nominal values.

**Initial pH**	**Final pH**	
	**SeO_3^{2-}**	**SeO_4^{2-}**
2	2.12	2.08
4	3.78	4.06
6	5.19	5.83
8	6.16	6.41
10	8.62	8.34

Section S3: Determination of Maximum Capacity of IRA-900 from Fixed-bed Experiments

Solutions of SeO_4^{2-} and SO_4^{2-} at concentrations of 50 mM were separately passed through a fixed-bed column packed with 5 g pristine IRA-900 at a flow rate of 30 mL/min for an hour to saturate the resin. During each set of experiments, all the effluent was collected and thoroughly mixed to accurately measure the average concentration of SeO_4^{2-} or SO_4^{2-}. To determine the maximum uptake capacity of the resin, liquid samples were collected from the feed solution, the mixed effluents, and the outlet of the fixed-bed column at the end of each experiment when saturation was reached. The maximum exchange capacity of IRA-900 from fixed-bed experiments was determined through Equation S1:

$$q_T = \frac{(c_0 - c_f) \cdot v \cdot t}{M} \tag{S1}$$

where q_T is the maximum exchange capacity, meq/g; c_0 and c_f are the initial concentration of feed solution and the final (outlet) concentration after passing through the fixed-bed column, respectively, meq/L; v is the flow rate, L/min; t is the total operational time, min; M is the total resin mass packed in the fixed-bed column, g.

The total ion exchange capacity (q_T), or theoretical exchange capacity, refers to the mass of exchangeable ions per unit mass of resin and indicates the theoretical number of available exchange sites. This parameter is important for evaluating resin performance and providing the theoretical maximum exchange capacity as an input to the models. For IRA-900, duplicate fixed-bed saturation experiments were conducted for both SeO_4^{2-} and SO_4^{2-}, with results shown in Table S2. The average capacities were 2.04 ± 0.04 meq/g for SeO_4^{2-} and 2.05 ± 0.35 meq/g for SO_4^{2-}. While both values are consistent and center around ~2.0 meq/g, the higher standard deviation for SO_4^{2-} reflects some experimental variability. Remarkably, although the batch experiments showed

a higher affinity of IRA-900 for SeO_4^{2-} than for SO_4^{2-}, the fixed-bed experimental results indicate that both ions ultimately reach identical maximum capacities. In addition, the exclusion of SeO_3^{2-} from this measurement is due to its significantly lower affinity compared to that for SeO_4^{2-} and SO_4^{2-}, which would result in an inaccurate determination of the resin's maximum exchange capacity.

Table S2. Total ion exchange capacity (q_T) of IRA-900 determined from fixed-bed saturation experiments with SeO_4^{2-} and SO_4^{2-}.

Oxyanion	Experiment	q_T (meq/g)	Average q_T (meq/g)
SeO_4^{2-}	Run 1	2.06	2.04 ± 0.04
	Run 2	2.01	
SO_4^{2-}	Run 1	2.29	2.05 ± 0.35
	Run 2	1.80	

Section S4. Optimized Equilibrium and Kinetic Parameters for LMA and Transport Modeling

Table S3. Optimized equilibrium parameters for Cl^-/SeO_4^{2-} and Cl^-/SO_4^{2-}; results were obtained by optimizing parameters for both the ideal and non-ideal LMA models.

System	Non-ideal LMA	Ideal LMA
	$K_{Cl^-}^{SeO_4^{2-}} = 186.47$	
Cl^-/SeO_4^{2-}	$\Lambda_{Cl^-}^{SeO_4^{2-}} = 1.47$	$k_{Cl^-}^{SeO_4^{2-}} = 201.05$
	$\Lambda_{SeO_4^{2-}}^{Cl^-} = 0.68$	
	$K_{Cl^-}^{SO_4^{2-}} = 52.27$	
Cl^-/SO_4^{2-}	$\Lambda_{Cl^-}^{SO_4^{2-}} = 1.63$	$k_{Cl^-}^{SO_4^{2-}} = 54.95$
	$\Lambda_{SO_4^{2-}}^{Cl^-} = 0.61$	
	$K_{Cl^-}^{SeO_3^{2-}} = 2.88$	
Cl^-/SeO_3^{2-}	$\Lambda_{Cl^-}^{SeO_3^{2-}} = 1.31$	$k_{Cl^-}^{SeO_3^{2-}} = 1.08$
	$\Lambda_{SeO_3^{2-}}^{Cl^-} = 0.76$	

Table S4. Optimized kinetic parameters for (a) SeO_4^{2-}, (b) SO_4^{2-}, and (c) SeO_3^{2-} uptake experiments operated at different initial concentrations. In the tables below, symbol q denotes the resin phase capacity, and symbol C_b denotes the concentration of oxyanion in bulk solution as shown in Figure 9 in the main manuscript.

(a)

**Parameters**	**Initial concentration ($\times 10^{-3}\,\frac{meq}{mL}$)**		
	**2.72**	**11.23**	**38.24**
$k_f\ (\frac{\mathrm{meq}}{\mathrm{g}} \times \frac{\mathrm{meq}}{\mathrm{mL}})^{-1}$	5.03	5.18	5.06
$k_r\ (\frac{\mathrm{meq}}{\mathrm{g}} \times \frac{\mathrm{meq}}{\mathrm{mL}})^{-1}$	1.77E-03	1.31E-03	2.98E-03
$K = \frac{k_f}{k_r}$	2848.62	3962.58	1696.99
$k_m\ (\frac{\mathrm{cm}}{\mathrm{s}})$	5.99E-04	13.4E-04	9.44E-04
$D_p\ (\frac{\mathrm{cm}^2}{\mathrm{s}})$	1.30E-05	1.33E-05	1.93E-05
$\mathrm{R}^2(q_{\mathrm{SeO_4^{2-}}})$	0.99	0.99	0.96
$\mathrm{R}^2(C_{b,\mathrm{SeO_4^{2-}}})$	0.99	0.99	0.99

(b)

**Parameters**	**Initial concentration ($\times 10^{-3}\,\frac{meq}{mL}$)**		
	**2.08**	**9.98**	**20.02**
$k_f\ (\frac{\mathrm{meq}}{\mathrm{g}} \times \frac{\mathrm{meq}}{\mathrm{mL}})^{-1}$	10.02	10.15	10.09

$k_r\ (\frac{\text{meq}}{\text{g}} \times \frac{\text{meq}}{\text{mL}})^{-1}$	1.70E-02	0.48E-02	1.05E-02
$K = \frac{k_f}{k_r}$	589.40	2111.05	964.65
$k_m\ (\frac{\text{cm}}{\text{s}})$	1.12E-03	2.17E-03	47.1E-03
$D_p\ (\frac{\text{cm}^2}{\text{s}})$	7.32E-06	2.45E-06	19.3E-06
$\text{R}^2(q_{\text{SO}_4^{2-}})$	0.91	0.97	0.98
$\text{R}^2(C_{b,\text{SO}_4^{2-}})$	0.91	0.97	0.98

(c)

**Parameters**	**Initial concentration ($\times 10^{-3}\ \frac{\text{meq}}{\text{mL}}$)**		
	**1.90**	**10.36**	**20.00**
$k_f\ (\frac{\text{meq}}{\text{g}} \times \frac{\text{meq}}{\text{mL}})^{-1}$	5.48	5.45	4.73
$k_r\ (\frac{\text{meq}}{\text{g}} \times \frac{\text{meq}}{\text{mL}})^{-1}$	3.01E-02	2.73E-02	9.47E-02
$K = \frac{k_f}{k_r}$	182.23	199.98	49.97
$k_m\ (\frac{\text{cm}}{\text{s}})$	5.60E-04	5.20E-04	81.9E-04
$D_p\ (\frac{\text{cm}^2}{\text{s}})$	49.8E-06	8.26E-06	3.32E-06
$\text{R}^2(q_{\text{SeO}_3^{2-}})$	0.99	0.99	0.98
$\text{R}^2(C_{b,\text{SeO}_3^{2-}})$	0.99	0.99	0.98

Section S5. High-Resolution XPS Analysis of IRA-900 Before and After Oxyanion Loading

Distinct Se 3d peaks were observed for the SeO_3^{2-}- and SeO_4^{2-}-loaded resins, whereas only a single intermediate peak appeared in the mixture-loaded resin due to overlap of Se (IV) and Se (VI) peaks (Figure S2). These results confirm ion exchange with Cl^- as the removal mechanism with no evidence of redox transformation.

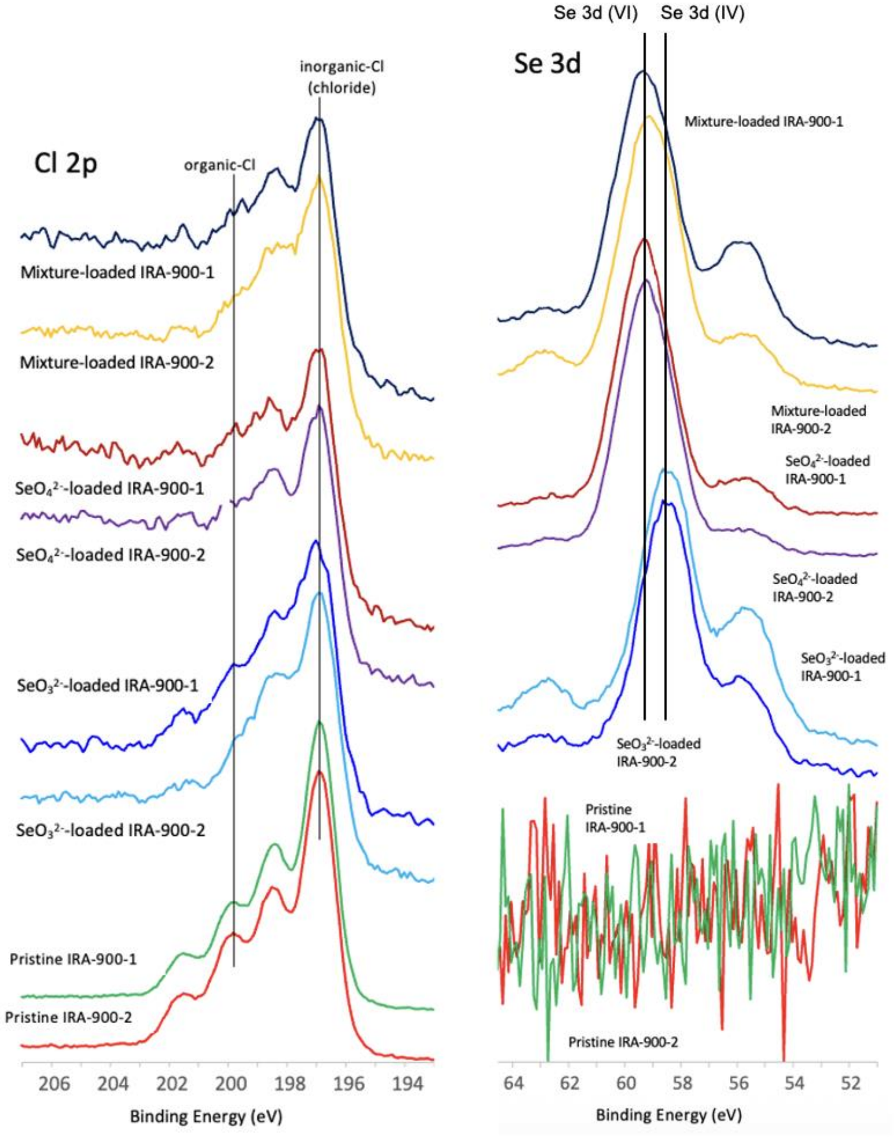

Figure S2. High-resolution XPS spectra of Cl 2p (left) and Se 3d (right) regions for pristine IRA-900 and after loading with SeO_3^{2-}, SeO_4^{2-}, or mixed solutions.
